# SCF-SKP2 E3 ubiquitin ligase links mTORC1/ER stress/ISR with YAP activation in murine renal cystogenesis

**DOI:** 10.1172/JCI153943

**Published:** 2022-12-15

**Authors:** Dibyendu K. Panda, Xiuying Bai, Yan Zhang, Nicholas A. Stylianesis, Antonis E. Koromilas, Mark L. Lipman, Andrew C. Karaplis

**Affiliations:** 1 Division of Endocrinology and Metabolism, Department of Medicine, and Lady Davis Institute for Medical Research, Jewish General Hospital,; 2 Division of Nephrology, Department of Medicine, and Lady Davis Institute for Medical Research, Jewish General Hospital,; 3 Department of Chemical Engineering, Faculty of Engineering, and; 4 Gerald Bronfman Department of Oncology, Faculty of Medicine, McGill University, Montreal, Quebec, Canada.; 5 Lady Davis Institute for Medical Research, Jewish General Hospital, Montreal, Quebec, Canada.

**Keywords:** Nephrology, Chronic kidney disease

## Abstract

The Hippo pathway nuclear effector Yes-associated protein (YAP) potentiates the progression of polycystic kidney disease (PKD) arising from ciliopathies. The mechanisms underlying the increase in YAP expression and transcriptional activity in PKD remain obscure. We observed that in kidneys from mice with juvenile cystic kidney (*jck*) ciliopathy, the aberrant hyperactivity of mechanistic target of rapamycin complex 1 (mTORC1), driven by ERK1/2 and PI3K/AKT cascades, induced ER proteotoxic stress. To reduce this stress by reprogramming translation, the protein kinase R–like ER kinase–eukaryotic initiation factor 2α (PERK/eIF2α) arm of the integrated stress response (ISR) was activated. PERK-mediated phosphorylation of eIF2α drove the selective translation of activating transcription factor 4 (ATF4), potentiating YAP expression. In parallel, YAP underwent K63-linked polyubiquitination by SCF S-phase kinase-associated protein 2 (SKP2) E3 ubiquitin ligase, a Hippo-independent, nonproteolytic ubiquitination that enhances YAP nuclear trafficking and transcriptional activity in cancer cells. Defective ISR cellular adaptation to ER stress in eIF2α phosphorylation–deficient *jck* mice further augmented YAP-mediated transcriptional activity and renal cyst growth. Conversely, pharmacological tuning down of ER stress/ISR activity and SKP2 expression in *jck* mice by administration of tauroursodeoxycholic acid (TUDCA) or tolvaptan impeded these processes. Restoring ER homeostasis and/or interfering with the SKP2-YAP interaction represent potential therapeutic avenues for stemming the progression of renal cystogenesis.

## Graphical abstract

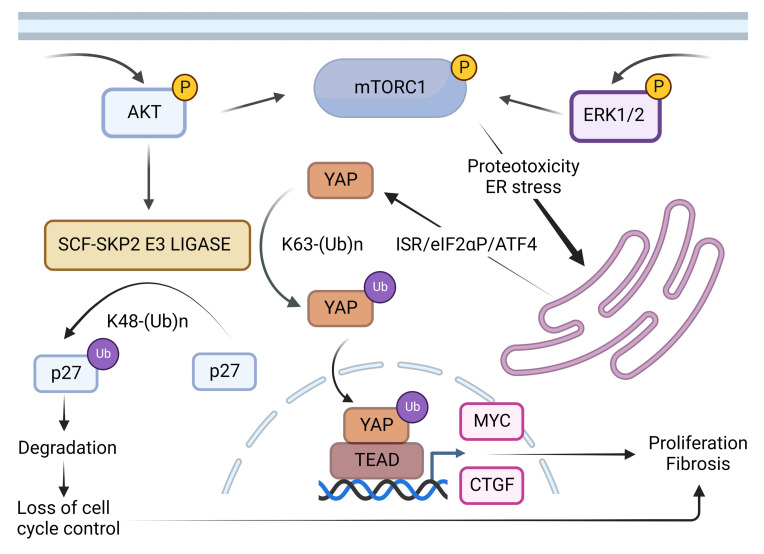

## Introduction

The primary cilium is a single, nonmotile sensory organelle present at the cell surface. It senses mechanical and chemical environmental cues and conveys these external signals to the cell’s interior (1). Linked fundamentally to the mitotic cell cycle, the primary cilium orchestrates organ development and tissue homeostasis.

In the kidney, the primary cilium in tubular epithelial cells localizes to the apical surface and translates physical events to intracellular signals that modulate cellular functions (2). As genomic and genetic approaches identify novel mutations in genes encoding proteins critical for ciliary function, the number of ciliopathies continues to expand (3). Inherited ciliopathies lead to the development of renal cysts, ultimately causing kidney damage and end-stage kidney disease (ESKD). These ciliopathies encompass autosomal dominant polycystic kidney disease (ADPKD), the most common genetic renal disease and an important cause of ESKD, autosomal recessive polycystic kidney disease (ARPKD), and nephronophthisis (NPH), a genetically heterogeneous group of kidney diseases representing the most common genetic cause of ESKD in children.

*PKD1* and *PKD2* mutated in ADPKD encode for the major ciliary-associated proteins polycystin 1 (PC1) and polycystin 2 (PC2), respectively (4, 5). PC1 is a large transmembrane protein, whereas PC2 is a member of the transient receptor potential (TRP) superfamily. PC2 colocalizes with PC1 and functions to regulate intracellular Ca2^+^ levels and cell growth (6). In addition, PC2 is found in the same complex as protein kinase R-like ER kinase (PERK, gene name *EIF2AK3*) and eukaryotic translation initiation factor 2α (eIF2α, gene name *EIF2A*) on the ER membrane (7). The PERK/eIF2α arm is a critical component of the integrated stress response (ISR) and plays a major role in the survival and adaptation of cells to stress by repressing global protein synthesis via PERK-mediated phosphorylation of eIF2α (8, 9).

Loss of PC1 or PC2 leads to hyperproliferation of the tubular epithelial cells, giving rise to cystogenesis and massive kidney enlargement (10). In contrast, decreased proliferation and apoptosis exemplify the NPH group of diseases, a discrepancy ascribed in part to altered Hippo signaling, an evolutionarily conserved pathway that regulates cell proliferation and death, and hence organ size (10). In mammals, it is composed of the mammalian Ste20–like kinases 1 and 2 (MST1/2) and large tumor suppressor 1 and 2 (LATS1/2) kinase cassettes that act to phosphorylate and inhibit the transcriptional regulator Yes-associated protein (YAP) and its paralog transcriptional coactivator with PDZ-binding motif (TAZ). MST1/2 phosphorylates LATS1/2, which in turn induces phosphorylation of the effectors YAP (at S127) and TAZ, targeting them for sequestration by 14-3-3 in the cytoplasm. Alternatively, phosphorylation at other sites leads to YAP/TAZ K48-linked polyubiquitination by SCFβ-TRCP E3 ubiquitin (Ub) ligase and degradation by the proteasome (reviewed in ref. 11). Decreased Hippo signaling results in YAP/TAZ dephosphorylation and translocation to the nucleus, primarily via importin 7 (12), a process crucial for their functionality as transcriptional coactivators. There, YAP/TAZ binds to transcriptional enhanced associate domain (TEAD) transcription factors and activates YAP and TAZ target gene expression to promote cell proliferation, differentiation, and survival, with one of the major transcriptional target genes being MYC proto-oncogene (*MYC*) (13, 14). YAP/TAZ nucleocytoplasmic shuttling and MYC have been widely implicated in the pathogenesis of cystic kidney disease (13–15), as YAP expression and transcriptional activity increase in *Pkd1*-null mouse kidneys and human ADPKD tissues (14, 16). In this context, it is proposed that ADPKD and ARPKD arise from decreased LATS1/2 phosphorylation and increased YAP/TAZ transcriptional activity, while, conversely, increased LATS1/2 signaling and reduced cellular proliferation underlie the NPH forms (reviewed in ref. 10). Whether unbalanced Hippo signaling is in fact responsible for these differences remains to be determined (17).

Multiple signaling pathways are implicated in the initiation and progression of ADPKD (14, 18, 19). As a master regulator of cell metabolism, mechanistic target of rapamycin complex 1 (herein referred to as mTOR) reprograms the metabolic changes needed for cells to exit the quiescent state and enter the cell cycle. The interplay between mTOR activity and the primary cilium in this process has been extensively studied, and inhibition of mTOR activity is a potential therapeutic tactic for the treatment of ADPKD. However, 2 large-scale, randomized clinical trials have failed to confirm the efficacy of this approach (20). Tolvaptan, a selective arginine vasopressin receptor 2 (AVPR2) antagonist that inhibits cAMP production in response to AVP, is presently the only drug approved for the treatment of ADPKD. However, its modest therapeutic efficacy and troublesome side effects (polyuria and rare cases of unexpected liver dysfunction) account for the study high drop-out rate (21, 22). In search of more effective therapeutics with fewer adverse events, it becomes paramount to understand how these diverse signaling pathways integrate to initiate and potentiate the cystogenic process.

Here, we show that in PKD, the prevailing increase in ER stress that ensues as a result of proteotoxicity activates the PERK/eIF2/ATF4 arm of the ISR, a branch of the unfolded protein response (UPR), raising YAP expression levels. Increased YAP nuclear transport and YAP/TEAD-mediated transcriptional activity unexpectedly arise from Hippo-independent, nonproteolytic YAP-K63–linked ubiquitination by the SCF–S-phase kinase–associated protein 2 (SKP2) E3 Ub ligase complex. Importantly, pharmacological tuning down of ER stress and the ISR leads to decreased YAP and SKP2 expression and impaired cyst progression, thereby highlighting the potential clinical utility of targeting this pathway in PKD.

## Results

### Renal epithelial cell proliferation, apoptosis, and fibrosis in jck kidneys.

The spontaneously arisen renal cystic mouse model, juvenile cystic kidney (*jck*), arises from a homozygous G448V substitution in the highly conserved regulator of chromosome condensation 1 (RCC1) domain of the nephronophthisis 9 (NPHP9, aka NEK8) protein (*Nek8^jck/jck^*, herein referred to as *jck*) (23), a protein critical for cell-cycle regulation and ciliary function (24). The *jck* mutation is transmitted in autosomal recessive mode, and contrary to other forms of NPH9 mutations and more like human ADPKD, cysts in *jck* kidneys are formed from multiple segments of the nephron, leading to a continual decline in renal function and death by 20 weeks of age (25). EGFR overexpression and mislocalization, increased cAMP levels, activation of the RAS/RAF/MEK/ERK MAPK pathway, and sexual dimorphism in the progression of the cystic disease, with more aggressive disease in male mice, have also been reported (26). The *jck* missense mutation leads to YAP expression, YAP nuclear shuttling, and upregulation of YAP target gene transcription (27), findings that parallel those in *Pkd1*-null mouse kidneys and human ADPKD tissues (16). The molecular mechanisms underlying these observations, however, remain under active investigation. Therefore, first we set out to further assess the utility of this rodent PKD model for uncovering key molecular mechanisms that partake in these YAP actions and ultimately in the process of renal cystogenesis.

Enlargement due to the formation of multiple cysts distributed throughout the entire organ is the hallmark of *jck* kidneys, a process discernable even at 1 month of age, and progressing with advanced age (Supplemental Figure 1A; supplemental material available online with this article; https://doi.org/10.1172/JCI153943DS1). This feature recapitulates the ADPKD phenotype and contrasts sharply with other forms of NPH in which the kidneys are small. We found that the expression of aquaporin 2 (AQP2), a member of a family of highly selective transmembrane water channels, was decreased (Supplemental Figure 1B) (28). Normally located at the apical membrane of principal cells in the collecting duct, AQP2 transports water across the cell to regulate urine concentration. Its decreased expression leads to impairment in urinary concentrating ability, a phenomenon also observed in ADPKD and related to the modification of the medullary architecture by the ongoing cystic changes (29).

Ki-67, a marker of G_1_ to M phase transition, showed marked differences in its expression (Supplemental Figure 1C). While most WT renal tubular epithelial cells were Ki-67 negative, reflecting their mainly quiescent G_0_ status, most cells lining the cysts in *jck* kidneys remained Ki-67 positive, indicating that these cells had not entered the G_0_ phase. Cyclin-dependent kinase 1 (CDK1) and MYC expression levels were also increased in *jck* kidney tissue (Supplemental Figure 1D), as in ADPKD, suggestive of the dysregulated cell cycle in these cells and increased cell proliferation.

In *jck* kidneys, we observed increased expression of molecular markers of renal interstitial damage (nestin [NES], vimentin [VIM]) (Supplemental Figure 1E); dedifferentiation (SRY-box transcription factor 2 [SOX2] and POU class 5 homeobox 1 [OCT4]) (Supplemental Figure 1F); and epithelial-mesenchymal transition (EMT) (VIM, actin α 2, smooth muscle [α*Sma*], intercellular adhesion molecule 1 [*Icam*], vascular cell adhesion molecule 1 [*Vcam*], and vitronectin [*Vtn*]) (Supplemental Figure 1G). Consequently, histological changes — notably increased interstitial collagen deposition and tissue fibrosis — were apparent (Supplemental Figure 1H).

We observed TUNEL-positive apoptotic cells among the cyst-lining cells, but they were more abundant among noncystic renal tubular epithelial cells (Supplemental Figure 1I). Similar tissue distribution was observed by immunostaining for the proapoptotic Bcl-2 family member BCL2-like 11 (BIM) (30). These results differ from previously reported studies in which tubular epithelial cells had few apoptotic cells (26). The discrepancy could be attributed to the age of the animals used in our study, 4-month-old animals in our case rather than the 2-month-old animals used in the study reported in ref. 26, with 4 months being a point in disease progression when cyst enlargement is sufficiently prominent to negatively affect normal kidney architecture (31).

Altogether, these results support the contention that, like in ADPKD, increased epithelial cell proliferation is a key hallmark of the *jck* kidney phenotype, contributing to cyst development, tubulointerstitial fibrosis, and apoptosis. These observations, along with the availability and ease of manipulation of this rodent model and the slow progression of PKD, validate this model’s suitability for deciphering the molecular mechanisms of renal cystogenesis, with relevance to human PKD pathology.

### Increased intracellular signaling, mTOR activity, and ER stress in jck kidneys.

**Figure 1:**
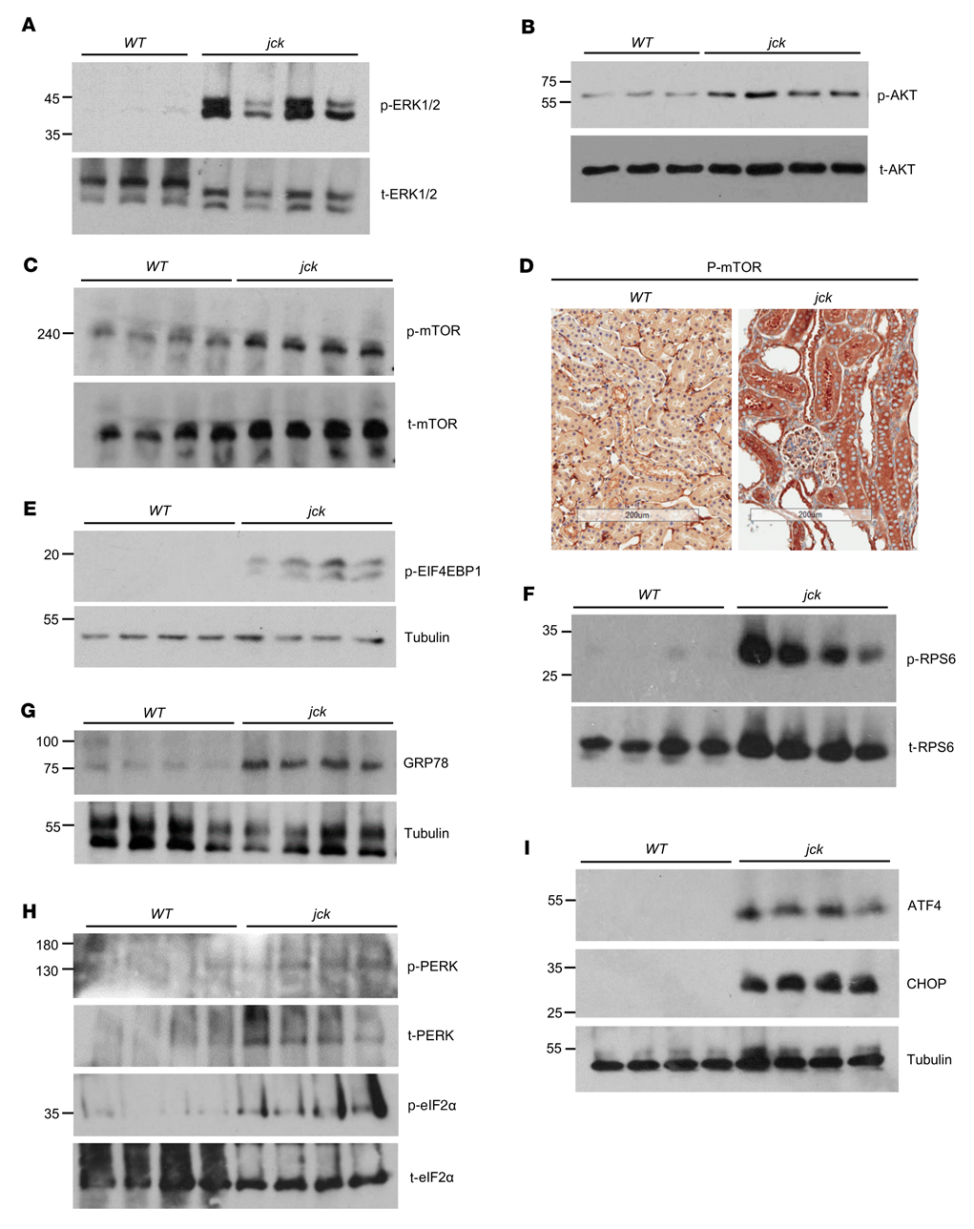
Aberrant mTOR activity, ER stress, and ISR in *jck* kidneys. Representative expression of (**A**) p-ERK1/2, t-ERK1/2, and (**B**) p-AKT and t-AKT in *jck* kidneys relative to WT expression levels. (**C**) p-mTOR and t-mTOR levels in WT and *jck* kidneys. (**D**) IHC images of p-mTOR expression. Scale bars: 200 μm. (**E**) Levels of the p-mTOR downstream target proteins EIF4EBP1 and (**F**) RPS6. (**G**) Increased GRP78 expression, consistent with increased ER stress in *jck* kidneys. (**H**) Activation of the PERK arm of the ISR (p-PERK) and p-eIF2α levels in *jck* kidneys. (**I**) Increased ATF4 and CHOP expression in *jck* kidneys compared with WT expression.

Next, we sought to validate in *jck* kidneys, in addition to MYC, the presence of other known mediators of the prevailing hyperproliferative state. In ADPKD, increased cAMP levels and activation of the RAS/RAF/MEK/ERK MAPK and AKT/mTOR pathways, mediated in part by AVPR2 signaling and high EGFR activation, have been reported (18). Indeed, levels of phosphorylated extracellular signal–regulated kinase 1/2 (p-ERK1/2) and protein kinase B (p-AKT) were increased in *jck* kidneys compared with WT kidneys (Figure 1, A and B), accompanied by a substantial rise in p-mTOR levels (Figure 1, C and D). mTOR positively controls protein synthesis through various downstream effectors, mainly by phosphorylating the eukaryotic translation initiation factor 4E binding protein 1 (EIF4EBP1) and the ribosomal protein S6 kinase B1 (RPS6KB1)/ribosomal protein S6 (RPS6). Indeed, we observed an increase in p-EIF4EBP1 and p-RPS6 levels in *jck* kidneys, consequent to the increased mTOR activity in the background of the *jck* mutation (Figure 1, E and F).

Prolonged mTOR activity and an excessive rise in protein translation lead to the accumulation of misfolded or unfolded proteins, which in turn gives rise to proteotoxicity, ER stress, and activation of the ISR (9). The ISR promotes both cellular repair and survival by reducing the load of unfolded proteins through attenuation of general protein synthesis, a mainly adaptive response initiated by the ER transmembrane receptor PERK, one of the 4 arms of the ISR and a branch of the UPR. PERK is a transmembrane kinase in the ER lumen, where it associates with the molecular chaperone heat shock protein family A (Hsp70) member 5 (HSPA5, aka GRP78 and BiP) (Figure 1G). Sensing the protein homeostasis (proteostasis) defect, GRP78 is titrated away from PERK by the unfolded proteins, allowing for the phosphorylation and activation of PERK to take place (Figure 1H, upper panels). p-PERK in turn mediates the phosphorylation of eIF2α at S52, which blocks general protein translation (Figure 1H, lower panels). Paradoxically, while reducing the protein load in the ER (32), p-eIF2α preferentially translates mRNAs encoding several short upstream open reading frames (uORFs), such as activating transcription factor 4 (*Atf4*), with the protein acting as a master transcription factor of stress-responsive genes to alleviate the stress, restore ER proteostasis, and promote adaptation and cell survival (Figure 1I). In *jck* kidneys, the expression of GRP78, having multiple functions in relieving ER stress and maintaining cell viability (33), is increased (Figure 1G), primarily due to the concomitant and prevailing high ATF4 expression that potentiates its transcription.

If homeostasis in the ER cannot be reestablished and the damage is irreversible, the ISR switches from the prosurvival to the proapoptotic mode (34). ATF4 induces the expression of protein phosphatase 1 regulatory subunit 15A (PPP1R15A, aka GADD34), a mediator of eIF2α dephosphorylation that allows the restoration of general protein synthesis during prolonged ER stress, eventually leading to cell death. Also, the levels of the transcription factor DNA damage–inducible transcript 3 (DDIT3, aka C/EBP-homologous protein [CHOP]), implicated in ER stress–induced apoptosis, increase (35). Accordingly, we found that CHOP expression was markedly enhanced in *jck* kidneys but absent from WT kidneys, suggesting that the proapoptotic mode of the ISR was initiated (Figure 1I).

Taken together, these findings indicate that, in the context of the *jck* mutation, a state of hyperproliferation of the cyst-lining epithelial cells ensued, in part, via increased p-ERK1/2, p-AKT, and mTOR activity, recapitulating rather precisely the ADPKD phenotype (26). Under circumstances of relentless mTOR activation and ongoing ER stress, the proapoptotic mode of the ISR was triggered, ultimately increasing cellular dedifferentiation, collagen deposition, apoptosis, tissue damage, and fibrosis.

### YAP/TAZ expression and nuclear localization in jck kidneys.

**Figure 2:**
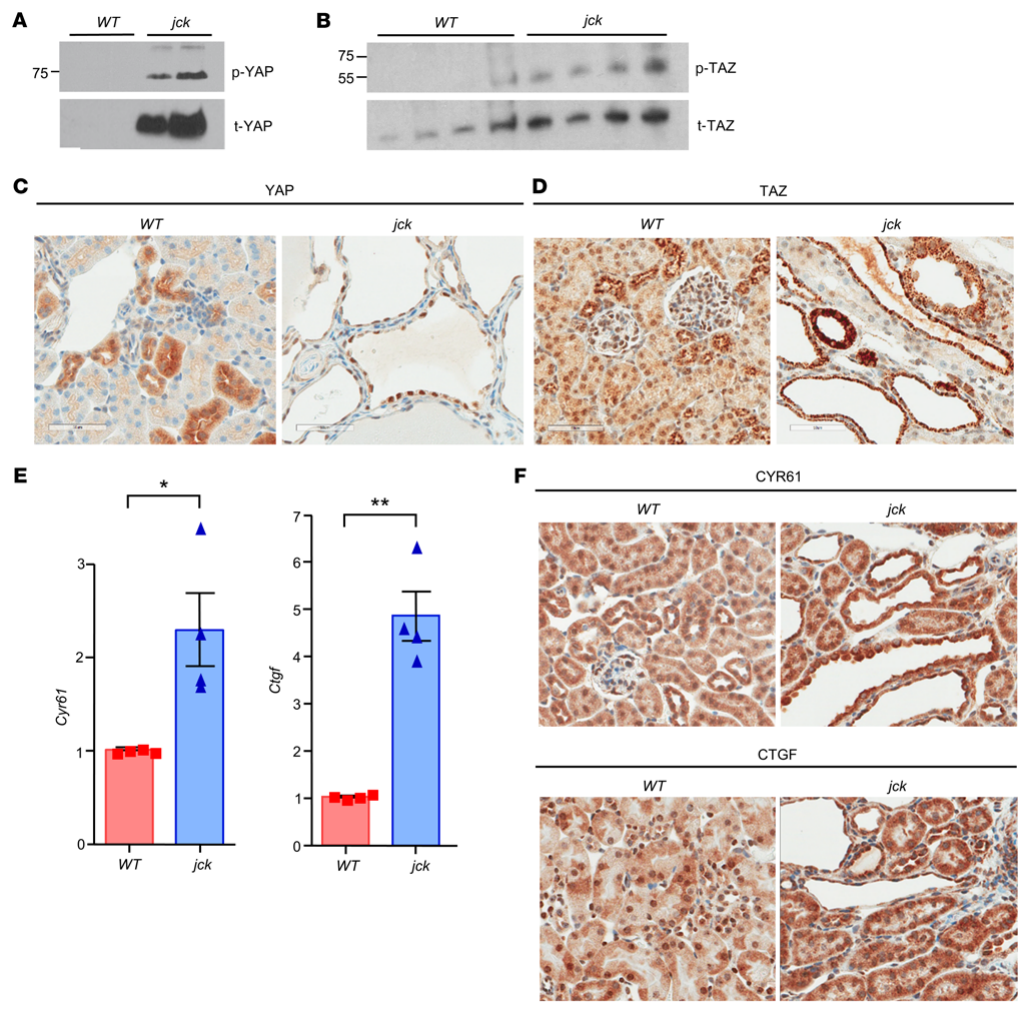
Increased YAP/TAZ expression in *jck* kidneys. (**A**) p**-**YAP and t-YAP expression in WT and *jck* kidneys. (**B**) p**-**TAZ and t-TAZ expression by Western blotting in WT and *jck* kidneys. (**C**) YAP and (**D**) TAZ expression by IHC in WT and *jck* kidney sections. Scale bars: 50 μm. (**E**) Expression of YAP and TAZ target genes *Cyr61* and *Ctgf* in *jck* kidneys compared with WT by quantitative real-time PCR and (**F**) by IHC. Data represent the mean ± SEM. *P < 0.05 and **P < 0.01, by unpaired Student’s *t* test.

The PERK/eIF2α/ATF4 arm of the ISR potentiates YAP expression, as ATF4 specifically binds to the YAP promoter following the induction of ER stress (36). Therefore, we next sought to determine whether the observed increase in ISR activity in *jck* kidneys was accompanied by comparable changes in the expression and transcriptional activity of the Hippo nuclear effectors YAP and TAZ. The levels of total YAP (t-YAP) and t-TAZ and p-YAP and p-TAZ were increased in *jck* kidneys compared with WT levels (Figure 2, A and B). Moreover, the ratios of p-YAP/t-YAP and p-TAZ/t-TAZ were small (more so for YAP), indicative of increased YAP/TAZ activity, as the phosphorylated forms are targeted for sequestration/degradation. While YAP was marginally detectable by Western immunoblots in WT kidneys, as previously reported (37), YAP staining using IHC was evident in several WT renal tubular epithelial cells and localized predominantly to the cytoplasm (Figure 2C). Immunostaining for YAP in *jck* kidney sections was more extensive, localizing almost exclusively to the nuclei of the cells lining the cysts, thereby substantiating its activation. In comparison, TAZ immunostaining in WT sections was both cytoplasmic and nuclear, and this pattern persisted in the epithelial cells lining the tubules and cysts in *jck* kidneys (Figure 2D).

The increased expression and nuclear localization especially of YAP would then be suggestive of increased transcriptional activity by the Hippo pathway effector. Indeed, the expression of the YAP/TAZ target genes cellular communication network factor 1 (*Ccn1*, aka *Cyr61*) and cellular communication network factor 2 (*Ccn2*, aka *Ctgf*) was also increased (Figure 2, E and F) in *jck* kidneys compared with WT, consistent with a rise in YAP-TAZ/TEAD transcriptional activity. Collectively, these observations indicate pronounced increases in YAP/TAZ expression and transcriptional activity in *jck* kidneys.

### An impaired ISR potentiates YAP activity and renal cystogenesis.

Although activation of the mTOR/ER stress/PERK/eIF2α/ATF4 pathway was evident in *jck* kidneys, its direct role in YAP activity and renal cystogenesis remains speculative. To this end, we next sought to evaluate its contribution by using the genetic approach. Specifically, the germline knockin mutation at the eIF2α phosphorylation site (eIF2α at S52A) (38) was introduced into the *jck* background, and renal cyst development was evaluated. The eIF2α (S52A) mutation decreases the phosphorylation of the α subunit of eIF2 and leads to chronic and unresolved ER stress by further augmenting general protein synthesis. Only mice heterozygous for the S/A-knockin mutation were used, as homozygotes die within 18 hours of birth (38).

**Figure 3:**
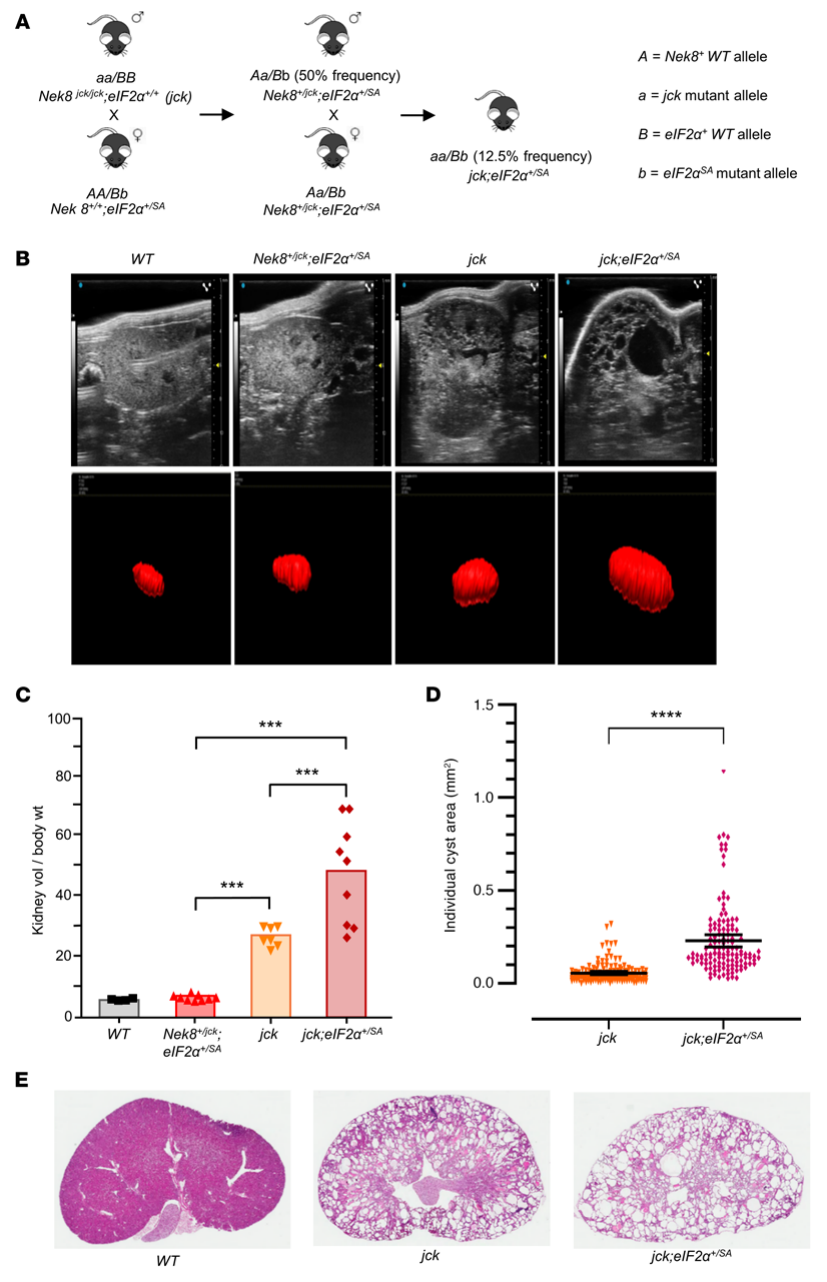
ER stress potentiates cystogenesis in *jck* mice. (**A**) Mice heterozygous for the SA-knockin mutation at the eIF2α phosphorylation site (eIF2α at S52A) were crossed onto the *jck* background. Mice of the 4 genotypes (WT, *Nek8^+/jck^* *eIF2α^+/SA^*, *Nek8^jck/jck^* *eIF2α^+/+^* [*jck*], and *Nek8^jck/jck^* *eIF2α^+/SA^*) were generated as shown schematically. The expected percentages of offspring with the indicated genotypes are shown. (**B**) Ultrasonographic examination of renal cysts at 3 months of age (top panels). Shown are representative images of kidneys generated by 3D reconstruction (red images, lower panels) used to determine relative kidney volume. (**C**) Kidney volume relative to body weight (wt) measurements. (**D**) Individual cyst area measurements (mm^2^) in kidneys from mice of the corresponding genotypes, as determined from ultrasonographic 2D images (*n* = 3 mice from each group). (**E**) H&E-stained kidney sections from mice of the indicated genotypes. Original magnification, ×10. Data represent the mean ± SEM. ***P < 0.001 and ****P < 0.0001, by 1-way ANOVA followed by a Tukey-Kramer multiple-comparison test for differences between the groups (**C**) and unpaired Student’s *t* test for comparison of the 2 groups (**D**).

Mice of the 4 genotypes — WT, *Nek8^+/jck^* *eIF2α^+/SA^* (double heterozygotes), *Nek8^jck/jck^* *eIF2α^+/+^* (*jck*), and *jck* *eIF2α^+/SA^* — were generated (Figure 3A), and renal cyst burden was determined by ultrasonography at 3 months of age (Figure 3B). The relative kidney volume/body weight ratio increased in the indicated genotype order, with the introduction of the hemizygous SA mutation into the *jck* background (*jck* *eIF2α^+/SA^*) fashioning the most impactful positive change on renal cystogenesis. The relative kidney volume/body weight ratio of mice heterozygous for both mutant alleles (*Nek8^+/jck^* *eIF2α^+/SA^*) was not different from that of WT mice (Figure 3, B and C). The mean individual cyst area was also significantly greater in *jck* *eIF2α^+/SA^* kidneys compared with *jck* (Figure 3D). Histologically, we observed multiple microscopic and macroscopic cysts affecting both the cortex and medulla (Figure 3E).

**Figure 4:**
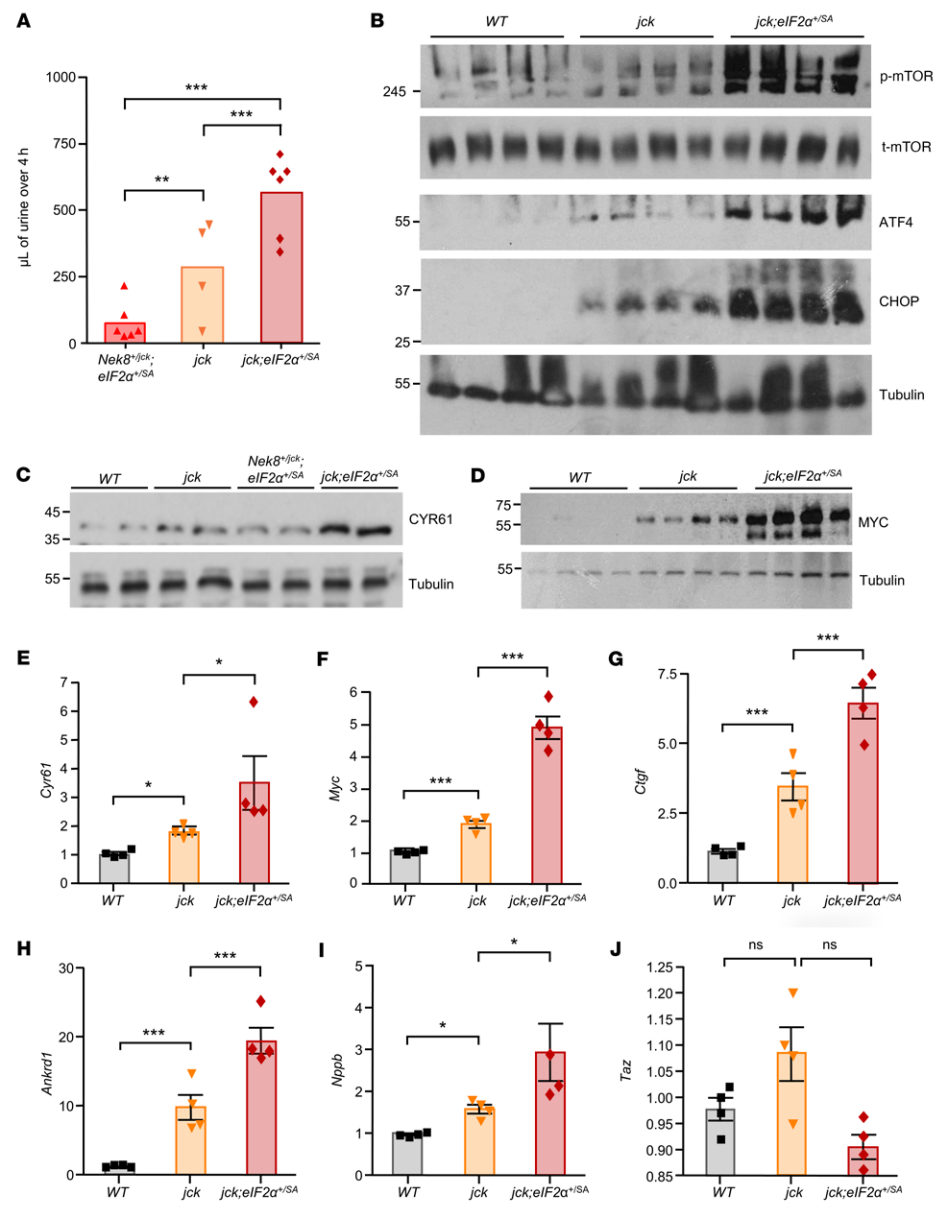
YAP transcriptional activity associates with renal cystogenesis. (**A**) Urine output (microliters of urine over 4 hours) by mice of the indicated genotypes. (**B**) Representative expression levels of p-mTOR and t-mTOR, ATF4, and CHOP in the kidneys of mice of the indicated genotypes (*n* = 4 mice for each genotype). (**C**) CYR61 and (**D**) MYC protein levels in kidneys from mice of the corresponding genotypes, as indicators of YAP target gene transcriptional activity (*n* = 2 and 4, mice respectively, for each of the indicated genotypes). mRNA expression of the YAP target genes (**E**) *Cyr61*, (**F**) *Myc*, (**G**) *Ctgf*, (**H**) *Ankrd1*, and (**I**) *Nppb* by quantitative real-time PCR relative to *Actb* in kidney extracts from mice of the indicated genotypes (*n* = 4 mice for each genotype). Data are representative of 2 independent experiments. (**J**) *Taz* mRNA expression in kidneys from mice of the indicated genotypes. Data represent the mean ± SEM. *P < 0.05, **P < 0.01, and ***P < 0.001, by 1-way ANOVA followed by a Tukey-Kramer multiple-comparison test.

These structural changes were also reflected in pathophysiological outcomes, as the *jck* *eIF2α^+/SA^* mice exhibited a significant increase in urine output compared with *jck* mice, likely due to the defective urine concentrating capacity arising from more aggressive tubular damage (Figure 4A) (39, 40). p-mTOR and markers of the ISR, ATF4 and CHOP, also increased significantly in *jck* *eIF2α^+/SA^* mice compared with *jck* mice (Figure 4B). p-eIF2α antagonizes mTOR activity (41), which may partly explain the higher levels of mTOR phosphorylation in *jck* *eIF2α^+/SA^* mice than in *jck* mice. Moreover, under stress, activated mTOR also stimulates the translation of ATF4 and CHOP (42, 43) and may contribute to the upregulation of both proteins in *jck* *eIF2α^+/SA^* mice, which have higher mTOR activity than *jck* mice.

We then questioned whether potentiation of mTOR/ER stress/eIF2α/ATF4 activity in response to the eIF2α mutant had affected YAP/TAZ transcriptional activity. Although the levels of CYR61 protein in *Nek8^+/jck^* *eIF2α^+/SA^* samples were comparable to those in WT samples, they increased in *jck* kidneys and became even more pronounced in *jck* *eIF2α^+/SA^* kidneys (Figure 4C). Similarly, MYC expression, while increased in the *jck* samples, was further potentiated by the single-allele eIF2α (S52A) mutation (Figure 4D). We observed parallel increases in *Cyr61* and *Myc* mRNA levels as well as in transcript levels of several other YAP-target genes including *Ctgf*, ankyrin repeat domain 1 (*Ankrd1*), and natriuretic peptide B (*Nppb*), whereas *Taz* expression, which is not a YAP target gene, was not significantly altered (Figure 4, E–J).

Therefore, the inability of the eIF2α^SA^-knockin mutation to effectively block protein translation arising from the prevailing mTOR activity ultimately potentiated ATF4 and CHOP expression. This in turn, augmented YAP transcriptional activity, leading to progressive renal cystogenesis and tissue damage.

### SCF-SKP2 E3 Ub ligase regulates YAP activity independent of Hippo.

**Figure 5:**
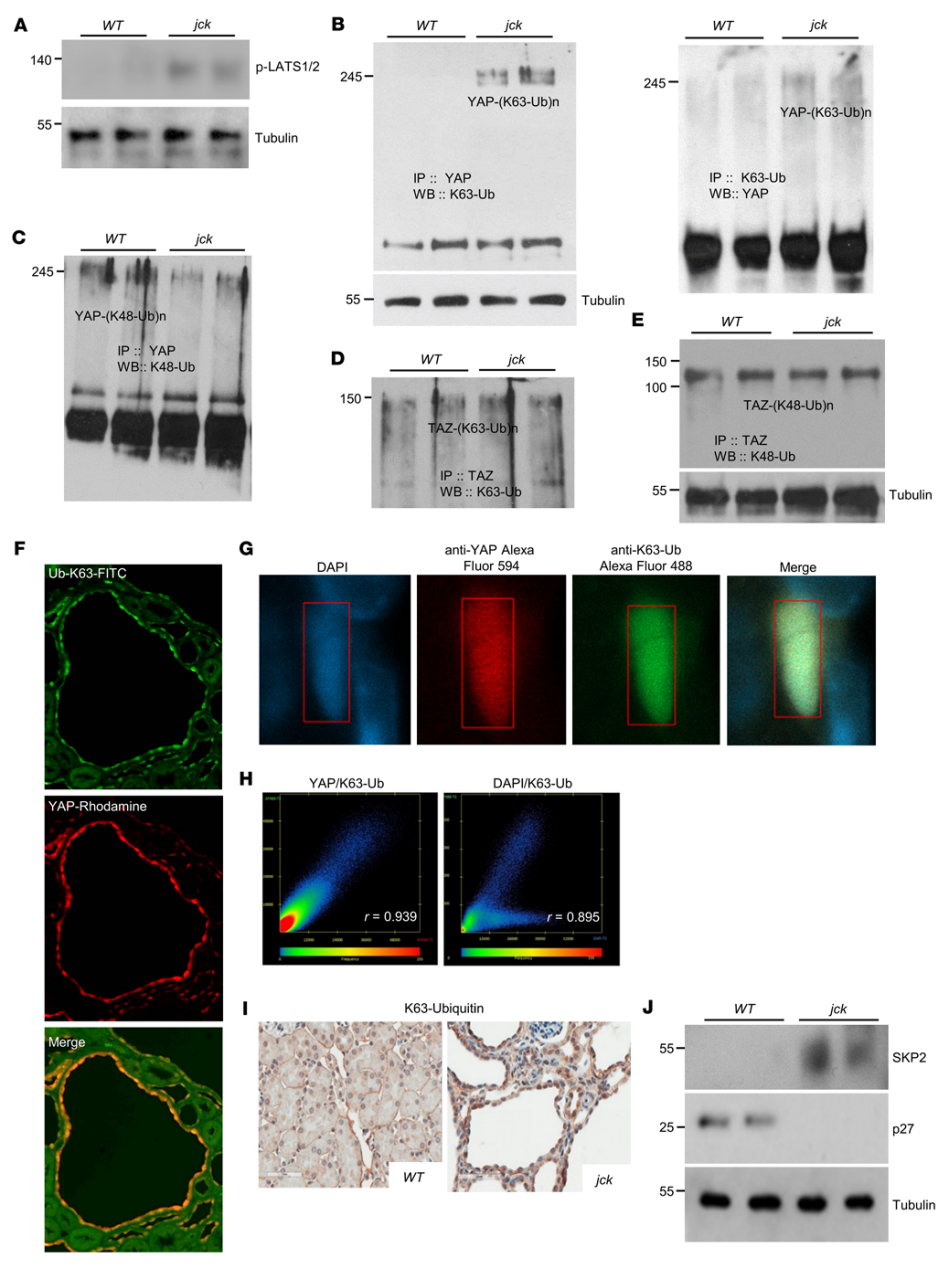
YAP K63-linked polyubiquitination and nuclear colocalization. (**A**) p-LATS1/2 expression in WT and *jck* kidney extracts. (**B**) t-YAP levels in kidney samples immunoprecipitated using a YAP antibody followed by immunoblotting with a K63-linked polyubiquitin-specific antibody (left panel). t–K63-Ub in kidney samples was immunoprecipitated using a K63-linked, polyubiquitin-specific antibody followed by immunoblotting with a YAP-specific antibody (right panel). (**C**) t-YAP in kidney samples was immunoprecipitated using a YAP antibody followed by immunoblotting with a K48-linked, polyubiquitin-specific antibody. (**D**) t-TAZ was immunoprecipitated using a TAZ antibody followed by immunoblotting with a K63-linked, polyubiquitin-specific antibody. (**E**) t-TAZ in kidney samples was immunoprecipitated using an antibody against TAZ followed by immunoblotting with a K48-linked, polyubiquitin-specific antibody. Results from 2 different kidney samples from mice of each of the 2 genotypes are shown. (**F**) Representative immunofluorescence micrographs of *jck* kidney sections showing K63-Ub and YAP colocalization in the nucleus. Upper panel: Immunostaining with a Ub-K63-FITC–specific antibody (green); middle panel: immunostaining with a YAP-specific rhodamine antibody (red); bottom panel: merged image of Ub-K63-FITC with YAP-rhodamine. Original magnification, ×20. (**G** and **H**) Laser-scanning confocal fluorescence microscopy of *jck* kidney sections using DAPI, anti-YAP Alexa Fluor 594, and anti–K63-Ub Alexa Fluor 488. The Pearson’s correlation coefficient (*r*) values, a measure of the strength of the linear relationship between 2 variables, YAP and K63-Ub in the left panel (*r* = 0.939) and DAPI and K63-Ub in the right panel (*r* = 0.895), are indicated. (**I**) Immunohistochemical localization of K63-linkage–specific ubiquitinated proteins in kidney sections. Scale bars: 50 μm. (**J**) SKP2 and p27 expression in WT and *jck* kidneys.

Although ATF4 augments YAP expression, we next questioned whether other signaling mechanisms could further potentiate YAP/TAZ actions. One obvious possibility is decreased Hippo signaling. We, therefore, sought to determine the status of LATS1/2 activity (phosphorylation) in *jck* kidneys. Unexpectedly, we observed higher levels of p-LATS1/2 in *jck* kidneys than in WT kidneys (Figure 5A), supporting neither our contention nor the prevailing paradigm (10). These results, while contrary to our expectations, were nevertheless consistent with our previous report that ATF4 stabilizes LATS1/2 (44).

How could we then reconcile these incongruent findings? Although the Hippo phosphorylation cascade causes cytoplasmic retention and inactivation of YAP, emerging evidence in cancerous cell lines suggests that YAP subcellular localization, and hence activity, are also regulated in a Hippo-independent manner (45). While polyubiquitination on defined lysine residues, notably on K48 and K29, is related to degradation by the proteasome, other polyubiquitinations (e.g., on K63, K11, K6, and M1) and monoubiquitinations regulate processes such as endocytic trafficking, inflammation, translation, and DNA repair (reviewed in ref. 46). In cancer cells, YAP was recently shown to undergo K63-linked polyubiquitination by SCF-SKP2 E3 Ub ligase at the K321 and K497 sites. This posttranslational modification, in sharp contrast to K48 polyubiquitination, potentiates YAP nuclear translocation and transcriptional activity (45).

To determine whether YAP was undergoing K63-linked polyubiquitination, we examined *jck* kidney extracts using an anti-YAP antibody for immunoprecipitation followed by Western immunoblotting with an anti–K63-Ub antibody. Although not a cancerous tissue, *jck*, but not WT, kidney extracts exhibited YAP K63-linked polyubiquitination (Figure 5B, left panel). We obtained similar results using the anti–K63-Ub antibody for immunoprecipitation and the anti-YAP antibody for immunoblotting (Figure 5B, right panel). In contrast, YAP immunoprecipitation followed by Western immunoblotting with an anti–K48-Ub–specific antibody revealed decreased K48-linked polyubiquitination of YAP in *jck* kidneys compared with the control (Figure 5C). A similar analysis for TAZ failed to demonstrate any differences in K63- or K48-linked ubiquitination between *jck* and WT kidney extracts (Figure 5, D and E). Subsequent studies therefore focused exclusively on YAP.

Fluorescence immunostaining suggested colocalization of YAP and K63-Ub in the nuclei of epithelial cells lining the cysts in *jck* kidneys (Figure 5F), and this was further validated using laser-scanning confocal fluorescence microscopy (Figure 5G). The Pearson’s correlation coefficient (*r*) for YAP/K63-Ub colocalization was 0.939, which is highly supportive of the proximity association for the 2 proteins (Figure 5H).

To follow up on these rather unexpected observations, we used an antibody against K63 linkage–specific polyubiquitin and observed prominent immunostaining in cells lining the renal cysts in *jck* mice, but substantially less immunostaining in WT tubular epithelial cells (Figure 5I). Since *SKP2* is reported to be a direct YAP target gene (47), we next examined SKP2 expression in *jck* and WT renal tissue extracts. SKP2 was undetectable in immunoblots of WT kidney extracts but highly expressed in *jck* (Figure 5J). SKP2 also mediates K48-linked polyubiquitination and degradation of the tumor suppressor cyclin-dependent kinase inhibitor 1B (CDKN1B, referred to herein as p27) that regulates G_0_ to S phase transition (48). Consistent with our expectations, p27 expression was present in WT kidneys but was undetectable in *jck*, given the prevailing high SKP2 levels.

In summary, these observations support the contention that, in the setting of ER stress/ISR/ATF4 activation, increased SKP2 expression ensues. One outcome is YAP’s nuclear relocation and transcriptional activity via K63-linked YAP polyubiquitination. In parallel, SKP2-mediated K48-linked polyubiquitination targets p27 for proteolysis, thereby contributing to the dysregulation of the cell cycle and the observed hyperproliferative state of the cyst lining cells.

### Deciliation increases ER stress and YAP activity.

Primary cilia are sensors on the cell membrane that sense surrounding mechanical and chemical signals. YAP is now recognized as a mediator of mechanical cues provided by the cellular microenvironment (49). The observed activation of YAP in human ADPKD and mouse *Pkd1* kidney cysts (14), and now in *jck* kidneys, raises the possibility that the sensing of external signals is linked to YAP regulation. To this end, we used Madin-Darby canine kidney II (MDCKII) cells that express a primary cilium (50) and first examined the role of laminar flow shear stress on YAP expression and transcriptional activity. Cells were plated in tissue culture plates and at the corresponding density in parallel-plate flow chambers (PPFCs) with either static culture medium fluid or under unidirectional flow for 12 hours at 3 dynes/cm^2^ laminar shear stress, maintained constant by the presence of an in-line dampener. We then assessed the expression of YAP, its target gene protein MYC, and other cell proliferation markers.

**Figure 6:**
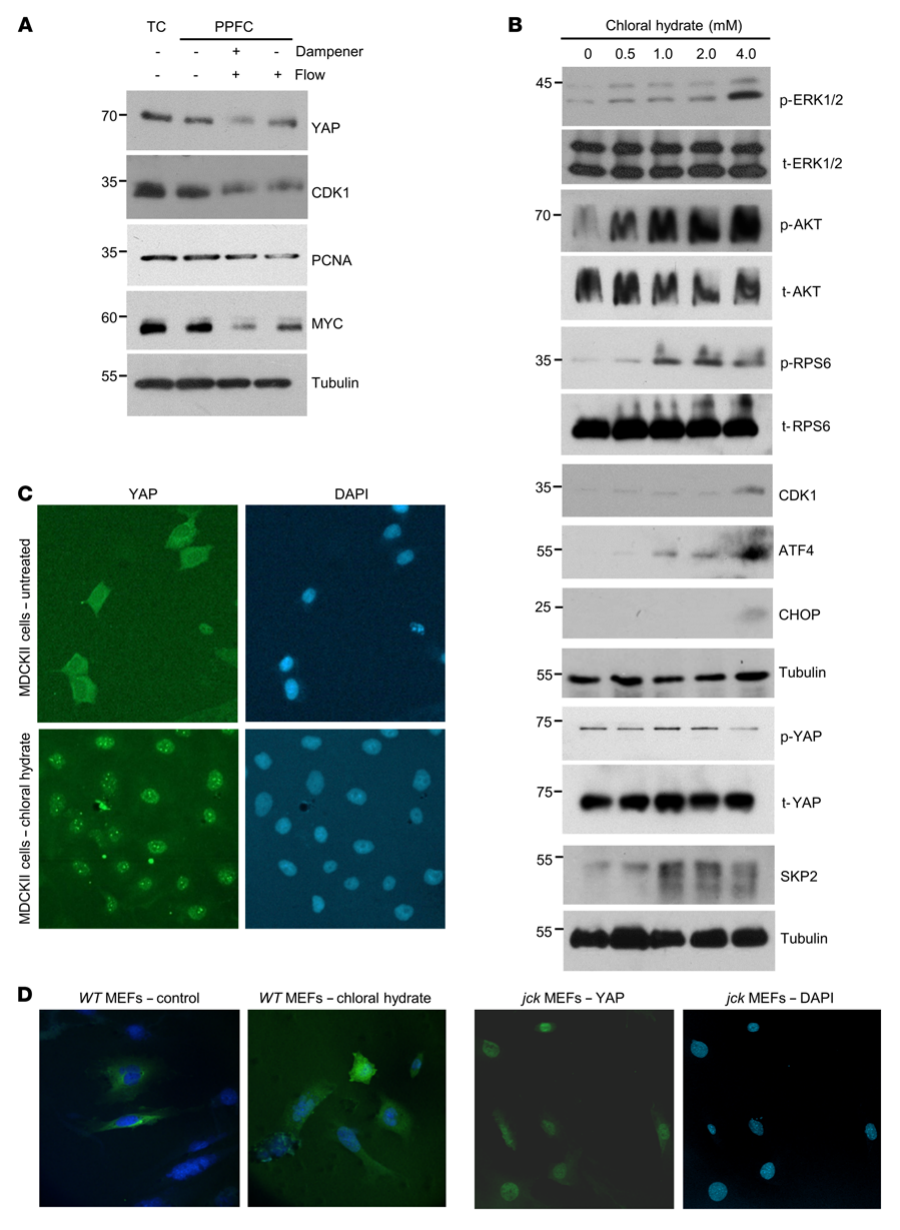
Deciliation induces ER stress, ATF4 and SKP2 expression, and YAP nuclear localization. (**A**) YAP, CDK1, PCNA, and MYC expression in MDCKII cells cultured either in a tissue culture (TC) dish or in a parallel-plate flow chamber (PPFC) unexposed or exposed to steady unidirectional laminar fluid shear stress (3 dyn/cm^2^) in the presence or absence of the dampener. (**B**) p-ERK1/2, t-ERK1/2, p-AKT/t-AKT, p-RPS6/t-RPS6, CDK1, ATF4, CHOP, p-YAP, t-YAP, and SKP2 expression levels following treatment of MDCKII cells with increasing concentrations of chloral hydrate to induce deciliation. (**C**) YAP immunofluorescence micrographs of MDCKII cells with or without chloral hydrate treatment (4 mM). DAPI fluorescence demarcates the nuclei. Original magnification, ×20. (**D**) YAP immunofluorescence in WT MEFs before and after chloral hydrate treatment (left panels) and in *jck* MEFs (right panels). Original magnification, ×20.

In the absence of flow, YAP levels in cells grown in the chamber were comparable to those in cells grown in tissue culture plates (Figure 6A). However, exposure to constant laminar flow markedly diminished YAP levels. In parallel, levels of the YAP target gene effector protein MYC declined, as did as those of the cellular proliferation markers CDK1 and proliferating cell nuclear antigen (PCNA) following exposure to fluid shear stress. These changes were partly mitigated when laminar flow shear stress was not constant, i.e., in the absence of a dampener.

The above-described changes may arise from the bending of the primary cilium due to flow (50). Alternatively, pressure on the cell surface by fluid flow may underlay these alterations (51). To further examine a link between loss of ciliary function and YAP activation, we assessed the molecular alterations that follow the deciliation of MDCKII cells using chloral hydrate (2). We observed that increasing concentrations of chloral hydrate potentiated the levels of p-ERK1/2, p-AKT, p-RPS6, and CDK1 (Figure 6B). In parallel, ATF4 and CHOP levels also increased following deciliation, while the ratio of p-YAP/t-YAP diminished, consistent with activation of YAP. Interestingly, chloral hydrate treatment also potentiated SKP2 expression in MDCKII cells.

Next, we examined the effect of deciliation on YAP subcellular relocalization in MDCKII cells using immunofluorescence. In untreated cells, YAP immunostaining localized mainly in the cytoplasm, but following treatment with chloral hydrate, it relocated almost exclusively to the nucleus (Figure 6C). Similar observations were made using mouse embryonic fibroblasts (MEFs) prepared from WT embryos (Figure 6D). In contrast, in MEFs derived from *jck* embryos, YAP immunofluorescence was mainly localized in the nucleus in the absence of chloral hydrate treatment.

Collectively, deciliation with chloral hydrate activated cellular signaling pathways that promoted ERK1/2 and AKT phosphorylation, mTOR activation, and potentiation of the ISR effectors ATF4 and CHOP, leading to YAP dephosphorylation, SKP2 expression, and YAP nuclear translocation.

### YAP inhibition impairs cystogenesis in an in vitro model.

Given the aforementioned observations, we next asked whether attenuation of ER stress impairs cyst formation. Evaluation of potential therapeutics targeting ER stress requires the availability of an in vitro model of cystogenesis to carry out the initial drug screening. Numerous in vitro models have been developed, each with its advantages and limitations (52). Here, we used the MDCKII 2D cyst-like model with forskolin stimulation (53, 54) and first assessed its utility as a screening tool.

Following stimulation of adenylate cyclase for 14 days with forskolin (10 μM) to increase intracellular levels of cyclic AMP, numerous multicellular 3D cystic-like structures appeared (Supplemental Figure 2A). As shown by immunofluorescence staining, strong YAP expression was detected in the cells lining these cyst-like structures. Cotreatment with verteporfin (1 μM), a potent YAP-TAZ/TEAD inhibitor blocking the transcription of target genes downstream of YAP (55), completely abolished the formation of these cystic structures (Supplemental Figure 2, B and C) and greatly reduced expression of the YAP/TAZ target genes *Ctgf* and *Cyr61* (Supplemental Figure 2D). In the presence of verteporfin, the levels of p-ERK1/2, p-mTOR, and p-RPS6 decreased over time, whereas t-ERK1/2, t-mTOR, and t-RPS6 levels were maintained. In parallel, YAP expression was also greatly reduced following verteporfin treatment (Supplemental Figure 2E).

Altogether, these findings indicate that, in this in vitro cystogenesis model, inhibition of YAP actions negatively affected parameters initiating and promoting the hyperproliferative state and cyst-forming capacity of forskolin treatment.

### Alleviation of ER stress impedes YAP activity in vitro.

Next, we assessed whether restoration of ER stress would also negatively affect cyst formation following forskolin treatment. The naturally occurring hydrophilic bile acid derivative tauroursodeoxycholic acid (TUDCA) is a taurine conjugate of ursodeoxycholic acid (UDCA), an FDA-approved drug for the treatment of primary biliary cholangitis. Several functions are ascribed to the action of TUDCA, primarily the alleviation of ER stress (56, 57). We therefore assessed the impact of TUDCA treatment on forskolin-induced cystic structure formation by cultured MDCKII cells.

Indeed, we found that TUDCA, in a concentration-dependent fashion, reduced the number the number of cyst-like foci that developed in the presence of forskolin (Supplemental Figure 3, A and B). The addition of TUDCA to the culture medium also decreased p-mTOR and p-RPS6 levels (Supplemental Figure 3C), which was probably a consequence of ER stress attenuation by TUDCA. Treatment of MDCKII cells for 24 hours with TUDCA progressively increased p-YAP levels (Supplemental Figure 3D). Accordingly, transcript levels of the YAP target genes *Ctgf* and *Cyr61* declined significantly (Supplemental Figure 3E).

Following treatment of MDCKII cells with forskolin, YAP immunofluorescence localized primarily in the nucleus. The concurrent addition of TUDCA, however, shifted its subcellular location, as YAP-associated green fluorescence became exclusively cytoplasmic (Supplemental Figure 3F).

We then questioned whether TUDCA would also alter YAP subcellular localization in response to AVP, a powerful potentiator of cystogenesis in vivo (58) via cAMP and the only approved therapeutic target for patients with ADPKD. Following treatment of MDCKII cells with AVP (10 nM), YAP immunofluorescence once again became almost exclusively nuclear. Yet, the concurrent addition of TUDCA relocalized YAP to the cytoplasm (Supplemental Figure 3G). Interestingly, treatment of MDCKII cells with AVP also increased SKP2 expression (Supplemental Figure 3H).

Finally, the selective AVPR2 antagonist tolvaptan, which blocks cAMP production in response to AVP, presently the only drug approved for the treatment of ADPKD (21), increased YAP phosphorylation in a dose-dependent manner in MDCKII cells (Supplemental Figure 3I).

In summary, these in vitro observations underscore the contention that restoration of ER homeostasis mitigates the formation of cyst-like structures in response to forskolin, in part by promoting molecular avenues that potentiate YAP phosphorylation and its cytoplasmic localization.

### Alleviating ER stress reduces renal cyst growth in jck mice.

On the basis of the aforementioned in vitro observations, we next questioned whether TUDCA would be as impactful as tolvaptan in vivo in slowing renal cyst progression in polycystic kidney disease (PKD) (59). We tested and compared in a 2-month trial the efficacy of these compounds individually using the *jck* murine model of PKD.

**Figure 7:**
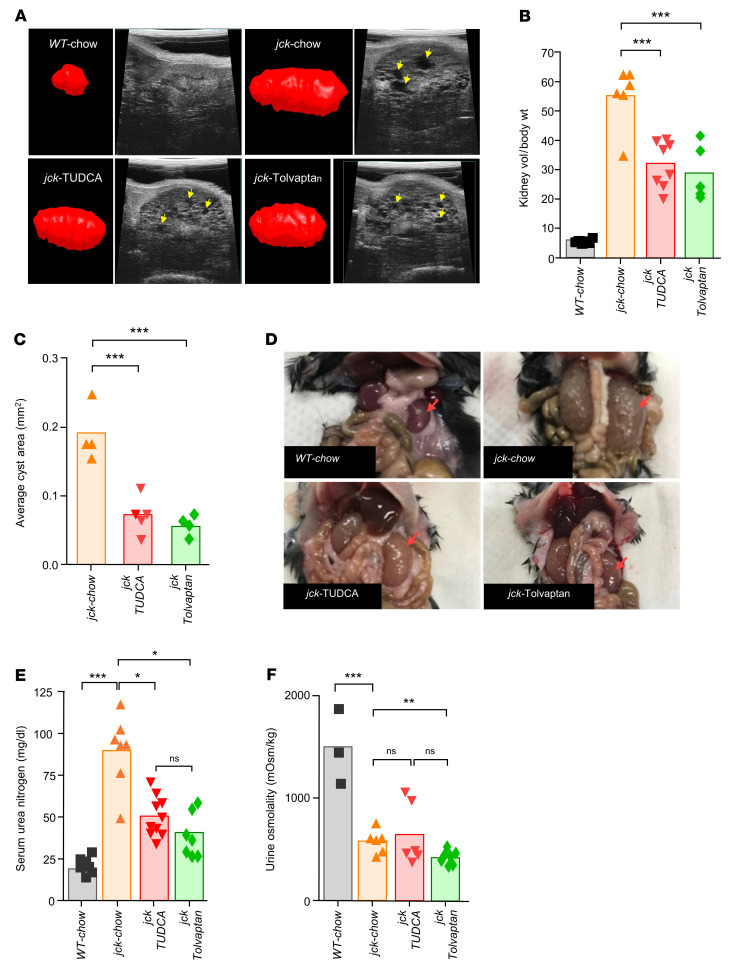
TUDCA and tolvaptan alleviate ER stress and slow renal cyst growth in *jck* kidneys. (**A**) In vivo ultrasonography of kidneys from WT and *jck* mice fed regular chow or chow containing TUDCA or tolvaptan. Yellow arrows indicate cysts. Shown in red are representative images of kidneys generated by 3D reconstruction used to determine the relative kidney volume. (**B**) Kidney volume/body weight ratio for WT, *jck*, and *jck* mouse treatment groups. (**C**) Average cyst area in mm^2^ in kidneys from mice in the control and 2 treatment groups. Cyst content was assessed from 2D images at the level where the renal artery could be identified. (**D**) Representative gross morphology of kidneys (arrows) procured from mice of each group at 3 months of age. (**E**) Serum urea nitrogen levels. (**F**) Urine osmolality following a 4-hour fast with access only to water. Each symbol represents an individual animal. Data represent the mean ± SEM. *P < 0.05, **P < 0.01, and ***P *<* 0.001, by 1-way ANOVA, followed by a Tukey-Kramer multiple-comparison test.

Starting at 1 month of age and for a period of 2 months, *jck* mice were treated with either TUDCA or tolvaptan introduced into the regular chow. Before euthanasia, we performed ultrasonography of the kidneys (Figure 7A) and measured kidney volume relative to body weight (Figure 7B). Both TUDCA- and tolvaptan-treated mice demonstrated a 40–50% reduction in the calculated ratio, which was associated with a 65%–75% decrease in the average cyst area (Figure 7C) and with the gross anatomic comparison of the ex vivo size of the procured kidneys (Figure 7D). Measurements of serum urea nitrogen levels demonstrated significant renal function preservation following treatment with TUDCA or tolvaptan compared with regular chow (Figure 7E). On the other hand, urine osmolality in the 3 *jck* groups was significantly decreased as a result of the urine-concentrating defect attributed to peripheral resistance to circulating AVP (60). Treatment with tolvaptan, however, trended toward a greater decrease, likely due to its additional inhibitory effects on AVP signaling (Figure 7F) (21).

**Figure 8:**
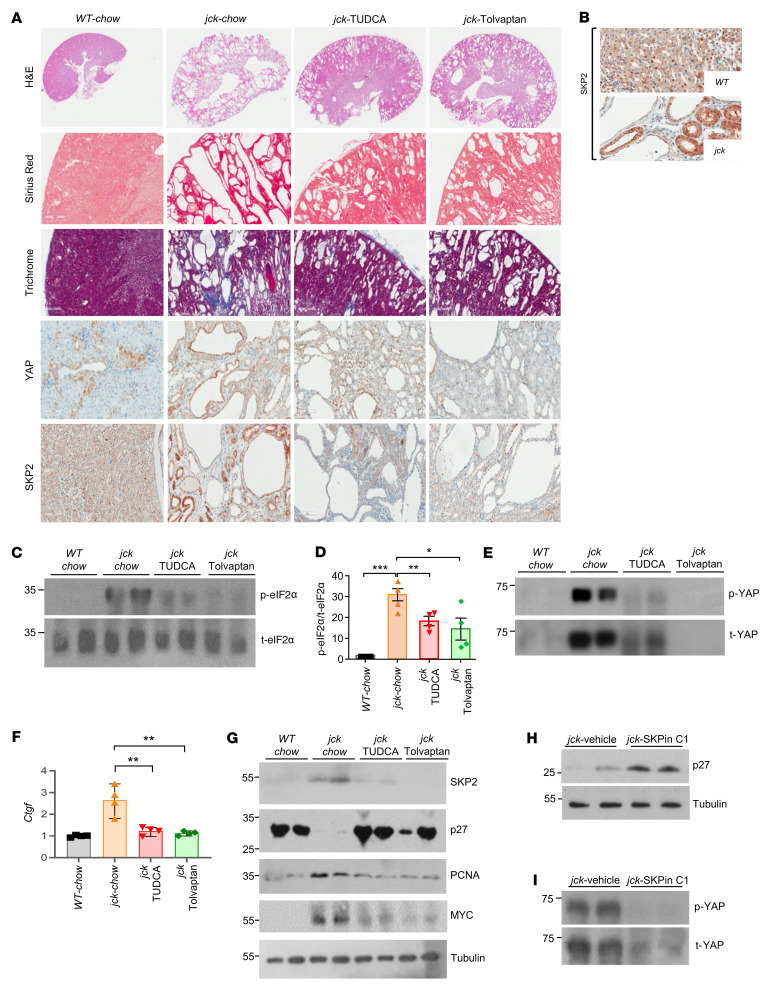
SKP2 plays a central role in renal cystogenesis. (**A**) Representative H&E, Picrosirius red (dark red indicating collagen), and trichrome (blue indicates fibrosis) staining and YAP and SKP2 immunostaining in kidney sections from mice in the control and treatment groups. (**B**) Higher magnification of SKP2 immunostaining in WT and *jck* kidneys demonstrating its differential subcellular distribution. Scale bars: 50 μm (**A**) and 100 μm (**B**). (**C**) Representative Western immunoblots for p-eIF2α/t-eIF2α. (**D**) Graphic representation of the quantified p-eIF2α/t-eIF2α ratio derived from Western blots using 4 kidney samples from mice in each of the groups. (**E**) Representative Western blot for p-YAP/t-YAP expression in kidneys from mice in the control and treatment groups. (**F**) Graphic representation of *Ctgf* expression using quantitative real-time PCR in the 4 kidney samples examined for each group using *Actb* as an internal control. (**G**) Representative Western blot analysis for SKP2, p27, PCNA, and MYC in kidneys from mice in each of the groups. (**H**) p27 and (**I**) p-YAP/t-YAP levels as determined by Western blotting in 2 representative kidneys from *jck* mice treated with vehicle or the SKP2 inhibitor SKPin C1. Each symbol in the graphs represents an individual animal. Data represent the mean ± SEM. *P < 0.05, **P < 0.01, and ***P < 0.001, by 1-way ANOVA followed by a Tukey-Kramer multiple-comparison test.

Histological assessment of kidney tissues further corroborated the ultrasonographic findings (Figure 8A). Indeed, the number and size of cysts were markedly reduced in response to either treatment. Most of the amelioration was evident in the medulla, although a modest improvement was also noted in the renal cortex of the treated mice. A reduction in collagen deposition and fibrosis was evident in the pericystic microenvironment in tissue sections from treated animals following staining with Picrosirius red and Masson’s trichrome, respectively. YAP immunostaining was drastically altered by TUDCA or tolvaptan treatment, as staining in cyst-lining epithelial cells was greatly diminished. In parallel, treatment with TUDCA or tolvaptan profoundly affected the expression of SKP2, as evidence by greatly reduced immunostaining. Notably, in *jck* sections, SKP2 cytoplasmic staining was more extensive, underscoring its pivotal role in promoting AKT activation (Figure 8B) (61).

To further decipher the mechanism by which these 2 disparate compounds positively affect the PKD phenotype, we investigated their effect on the PERK/eIF2α arm of the ISR. We found that the levels of p-eIF2α decreased following treatment with either drug, underscoring once more the central role of this pathway in renal cystogenesis (Figure 8, C and D). As anticipated, both drugs were effective in decreasing YAP expression in *jck* kidneys, as the levels of both t-YAP and p-YAP decreased following treatment (Figure 8E). Consequently, YAP activity was offset, as the levels of *Ctgf* transcripts normalized with either treatment (Figure 8F). In parallel, while increased SKP2 levels were observed in *jck* kidneys and were associated with the near-complete absence of p27 and higher PCNA and MYC expression levels consistent with the existing hyperproliferative state, treatment with either TUDCA or tolvaptan profoundly affected these parameters. SKP2 expression normalized, thereby corroborating the IHC findings (Figure 8G). Consequent to the decrease in SKP2, the expression of p27 and PCNA reverted to control levels, whereas MYC expression decreased following both treatments.

Overall, these results demonstrate that a major amelioration of the PKD phenotype resulted following TUDCA administration, similar to what was seen with tolvaptan. Both compounds profoundly affected ISR activity, ultimately decreasing YAP/SKP2 expression and YAP transcriptional actions.

### SKP2 inhibition restores p27 expression and impairs YAP activity.

The selective SKP2 inhibitor (SKPin) C1 is a small-molecule inhibitor of SKP2-mediated p27 degradation. It fits into a molecular surface pocket at the SKP2 interface with the adaptor protein CDC28 protein kinase regulatory subunit 1B (CKS1B), which is indispensable for the interaction and recognition of p27 by SKP2 and blocks p27 K48-linked ubiquitination (62).

One week after a single intraperitoneal injection of SKPin C1 into *jck* mice (63), p27 expression increased in whole-kidney lysates (Figure 8H). In parallel, the levels of p-YAP and t-YAP decreased, consistent with degradation (Figure 8I). These findings implicate SKP2 as a central contributor to cell-cycle dysregulation and YAP actions that potentiate the hyperproliferative and inflammatory/fibrotic consequences associated with renal cystogenesis (15).

### SKP2 expression increases following Pkd1 deletion.

Numerous rodent models have been developed for the study of ADPKD (52), yet no current model perfectly recapitulates the human disease. It is proposed, therefore, that any observation or ADPKD treatment strategy should be tested in at least 2 different animal models, 1 of which should be based on a *Pkd* mutation (64). Hence, we set out to determine whether increased SKP2 expression is observed in a tamoxifen-inducible, kidney epithelium–specific *Pkd1* deletion mouse model (65). Here, inactivation of *Pkd1* before P13 resulted in severely cystic kidneys within 3 weeks (early onset [EO]), whereas inactivation at day 14 and later resulted in cyst development only after 5 months (late onset [LO]).

**Figure 9:**
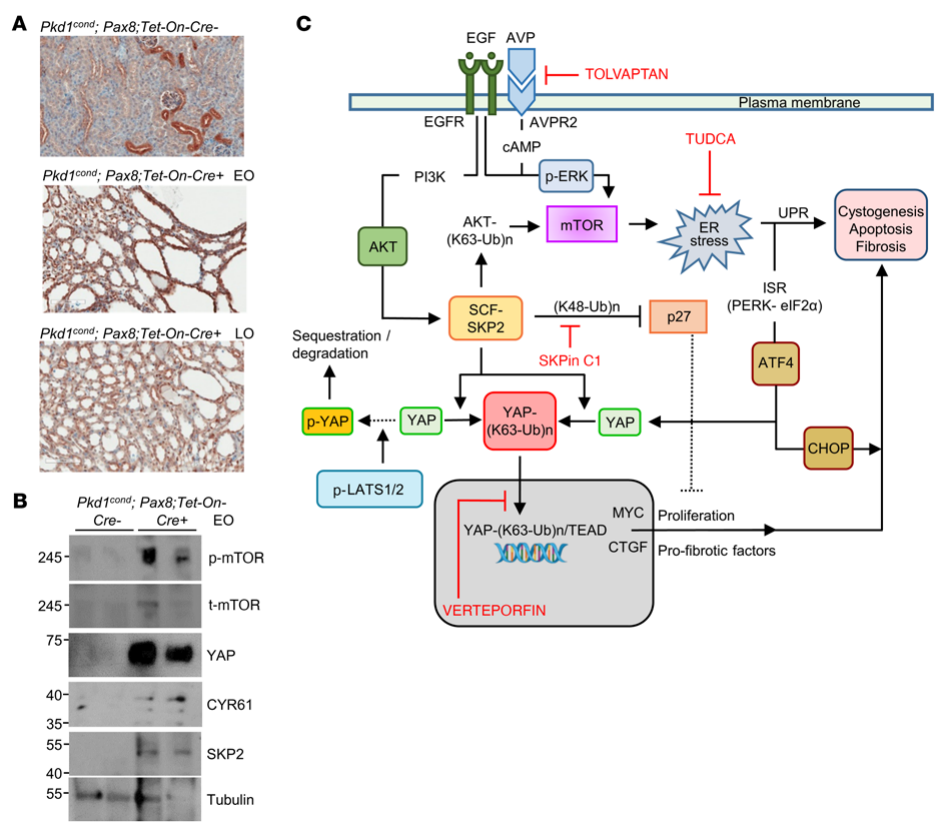
YAP and SKP2 expression in kidneys following *Pkd1* deletion. (**A**) YAP immunostaining in kidney sections from *Cre*^–^ (control) and *Cre^+^* EO and LO *Pkd1^cond^* *Pax8* *Tet-On^–^* mice. Scale bars: 100 μm. (**B**) p-mTOR, t-mTOR, YAP, CYR61, and SKP2 expression in kidney tissue extracts from *Cre^–^* and *Cre^+^* EO mice. (**C**) Schematic representation of the mechanistic pathways proposed to contribute to YAP-mediated renal cystogenesis and its progression. Lines represent direct/indirect activation (arrowhead) or inactivation (blunt end). The various drugs used for this study are indicated in red. Additional details are found in the body of the manuscript.

We first examined YAP expression in kidneys from *Cre^–^* and *Cre^+^* EO (induction P10–P11, termination P20) and LO (induction P27, termination P160) mice by IHC (Figure 9A). In the *Cre^–^* kidney, YAP immunostaining was prominent in some tubular epithelial cells, although it was exclusively cytoplasmic in its subcellular distribution. In contrast, in the *Cre^+^* EO kidneys, tubular epithelial cells displayed cytoplasmic and nuclear staining, while nearly all cyst-lining epithelial cells stained positive for nuclear YAP. In the *Cre^+^* LO sample, the majority of the tubular epithelial cells and most cyst-lining cells expressed YAP that localized predominantly to the nucleus. These findings support the contention that, in the absence of PC1, cystogenesis associates with YAP nuclear localization (65).

Next, we examined the phosphorylation status of mTOR and YAP and the expression of SKP2 in kidney tissue extracts from *Cre^–^* and *Cre^+^* EO mice (Figure 9B). The levels of p-mTOR increased in the absence of PC1 compared with the control, as did the levels of t-YAP. Upregulation of YAP transcriptional activity was further substantiated by the rise in CYR61 expression in *Cre^+^* EO kidney extracts. Last, immunoblots for SKP2 confirmed its expression in whole-kidney extracts from *Cre^+^* EO kidney but not in extracts from *Cre^–^* controls. Collectively, these observations support the applicability of our findings in *jck* kidneys to the ADPKD model, which closely recapitulated the molecular processes in the latter (Figure 9C).

## Discussion

The lack of effective treatment options for PKD calls for a fundamental reexamination of the molecular pathways that underpin the onset and progression of renal cystogenesis in this highly heterogeneous group of ciliopathies. Our findings uncovered several unexpected outcomes that we believe will have important clinical implications.

Ciliary dysfunction is a recognized cause of aberrant intracellular signaling (RAS/RAF/MEK/ERK, PI3K/AKT, and mTOR activation) (66). We now show that deciliation using chloral hydrate had similar effects on these molecular cascades, consequently inducing ER stress/ISR/ATF4 and SKP2 expression in kidney tubular epithelial cells, while promoting increased YAP levels and nuclear translocation. Ciliogenesis and the cell cycle are intimately linked, and our observations suggest that cilium-mediated mechanosensation of extracellular signals (for example, fluid shear stress arising from glomerular filtrate flowing on the surface of renal tubular cells) may perhaps be pivotal for halting renal epithelial cell proliferation. PC1, localizing largely in the primary cilium as well as in apical membranes, is proposed to act as a sensory molecule for fluid shear stress that transmits the signal from the extracellular fluid environment to PC2. The latter negatively regulates cell proliferation by enhancing PERK-dependent eIF2α phosphorylation (7, 67). Both molecules then provide baseline tonic inhibition of a signal effector that potentiates cyst growth when unchecked. We propose that one such major effector is YAP. In the absence of a functional sensor, aberrant intracellular signaling ensues, and YAP expression and its nuclear actions rise, ultimately potentiating cyst formation (68). Further studies will, however, be required to add support to or refute this supposition.

What underlies the increase in ER stress observed in PKD kidneys? In the setting of aberrant and uncontrolled EGFR and AVPR2 signaling, among others (69), high mTOR kinase activity prevails. The phosphorylation of EIF24EBP and RPS6K/RPS6 positively affects protein synthesis, as the former impedes translational inhibition, while the latter activates translation (70). The profound and relentless rise in protein translation has consequences, as it leads to proteotoxic ER stress. To reduce proteotoxicity, reprogramming of translation ensues through activation of the PERK/eIF2α arm of the ISR. General translation is shut off, and preferential translation of mRNAs such as the transcription factor ATF4 and its target gene products is initiated to restore ER homeostasis. In addition to this adaptive response, ATF4 translation is also activated by growth signals that stimulate mTOR, independent of the ISR and eIF2α phosphorylation (42, 71). The contribution of this pathway to the observed high ATF4 levels, however, remains to be determined.

It is in this background setting that we applied 2 experimental approaches to establish the pivotal role of the ER stress/ISR pathway in YAP action and ultimately in renal cystogenesis. The first was a genetic approach that introduced a heterozygous S/A-knockin mutation at the eIF2α phosphorylation site (eIF2α at S52A) (38) into the *jck* background. The inability to adequately shut down general protein synthesis in this animal model further augmented ER stress, YAP expression and activity, and renal cyst progression. The second was a pharmacological approach using the administration to *jck* mice of either TUDCA, which resolves ER stress and restores proteostasis, or tolvaptan, an AVPR2 antagonist and the only approved therapy for ADPKD. While the 2 drugs affect seemingly disparate signaling pathways, they both converged to downregulate the eIF2α/ATF4 arm of the ISR and decrease YAP expression and transcriptional activity. As such, our studies provide preclinical proof of principle that targeting ER stress is, in our view, a compelling, previously unrecognized therapeutic strategy for PKD.

Interestingly, in PKD kidneys, not only were the YAP levels increased but so was YAP transcriptional activity, all while the Hippo phosphorylation cascade that targets YAP for cytoplasmic sequestration and degradation was amplified. Here, in the PKD kidney, a noncancerous tissue, we report that YAP was subject to Hippo-independent, nonproteolytic K63-linked polyubiquitination by the SCF-SKP2 E3 ligase complex, as described in cancer cells. This complex is known to serve primarily as a central component of cell-cycle progression at the G_1_ phase by K48-linked ubiquitination and degradation of its primary substrate, p27 (72, 73). Apart from p27, SKP2 also initiates K48-linked ubiquitination and proteasome-mediated destruction of FOXO1, CARM1, p21, p57, and other mediators of cell-cycle regulation and diverse cellular functions (74). In addition to K48 ubiquitination of its proteolytic substrates, in cancerous cells, the SCF-SKP2 E3 ligase complex also mediates the K63-linked ubiquitination that modulates the function of its target substrates (74). Prominent in this group are AKT and YAP (45, 75). Via this process, the ligase complex promotes YAP-TEAD interaction, which in turn retains YAP in the nucleus (45), enhances its transcriptional activity (45), and contributes to the relentless hyperproliferation of cancerous cells. Our findings indicate that PKD cyst epithelial cells also utilize this process to increase YAP transcriptional activity and thereby potentiate cyst growth and the progression of tubulointerstitial fibrosis by *Myc* (13) and *Ctgf* (15) overexpression, respectively.

Several potential mechanisms could explain the increased levels of SKP2 in *jck* kidneys. First, the PI3K/AKT pathway, which is activated in this setting, increases *Skp2* transcription, while promoting SKP2 protein stability via phosphorylation (73). Upstream regulation of mTORC2 by the PI3K/AKT axis also contributes to SKP2 stability, as the mTORC2 kinase directly phosphorylates SKP2 and prevents its degradation (76). Finally, *SKP2* was identified recently as a YAP target gene (77), a pivotal observation that requires further consideration. Our demonstration that SKP2 levels were also increased in *Pkd1*-null kidneys points to the wider applicability of our findings in other forms of ciliopathy-related kidney cyst disease, as opposed to being viewed as a select consequence of the *jck* mutation.

It is of interest here to assess the commonalities between PKD and cancer, which are provocative (78). While PKD does not predispose to cancer development (79), the extensive similarities between PKD and solid tumors are intriguing, such that PKD is often referred to as “neoplasia in disguise” (80). The similarities, underscored primarily by sustained proliferative signaling, were reinforced further by our observations of SKP2-mediated YAP-K63 ubiquitination. Owing to its vital role in cell-cycle regulation, SKP2 plays a critical role in human cancers, where its overexpression is associated with poor survival and adverse therapeutic outcomes (74). In normal cells, SKP2 mainly localizes in the nucleus, while during cancer progression, SKP2 translocates to the cytoplasm, an observation that strikingly parallels its localization (primarily cytoplasmic) in *jck* kidney tubular epithelial cells. The phosphorylation of SKP2 by AKT is the molecular switch that critically controls the formation, localization, and function of the SKP2-SCF complex (61).

Based on the discussion above, we can now envision developing strategies to interfere with a number of these signaling pathways involved in the pathogenesis of PKD. Having now underscored the importance of the mTOR/ER stress/ISR pathway in the onset and progression of PKD, consideration should be given to using compounds such as TUDCA, either alone or in combination with EGFR or SRC inhibitors, or rapalogs (reviewed in ref. 81) for the treatment of PKD (82, 83). A growing number of preclinical and clinical studies highlight the potential benefit of this naturally occurring bile acid in a variety of ER stress–driven pathologies (84). Alternatively, SKPins, which are small-molecule inhibitors of SKP2-mediated p27 degradation (62) with potent antitumor activities in vitro and in preclinical mouse models (63), are highlighted in our studies as a potential therapeutic approach for the treatment of PKD. The mechanism by which restoration of p27 expression by SKPin C1 affects YAP phosphorylation remains to be determined. Studies aiming to address whether the inhibitor also interferes with SKP2-YAP interaction and/or YAP K63-linked polyubiquitination are ongoing. Nevertheless, given our findings, the development of small molecules that interfere with SKP2 action in general or specifically with SKP2-YAP interaction could serve as potential therapeutic approaches for PKD. In the future, it may also be of interest to investigate the therapeutic efficacy of these compounds in a combinatorial fashion.

While rodent models based on the engineering of *Pkd1* and *Pkd2* are an obvious choice to study the pathophysiological mechanisms of ADPKD, they present several limitations that hinder their utility (52). The *Nek8 jck* mutation is transmitted in autosomal recessive mode, yet it closely recapitulates the ADPKD phenotype (26). Our analysis of these mice here, in addition to previous reports (26, 37, 85), further validates their utility as a suitable rodent model for deciphering the molecular mechanisms of renal cystogenesis and for testing new therapies. These mice could therefore be viewed as a suitable murine PKD model for dissecting these mechanisms and a valuable preclinical tool for evaluating the efficacy and safety of novel therapeutics (85).

In conclusion, our findings provide the necessary backdrop for what we believe to be a previously unrecognized molecular mechanistic framework in PKD kidneys that could be targeted pharmacologically. Restoring ER homeostasis and/or interfering with SKP2-YAP interaction represent therapeutic approaches that could stem the progression of cyst growth in ciliopathy-related cystic kidney disease.

## Methods

Additional details on methods are provided in the Supplemental Methods.

### Mice and genotyping.

*jck* mice on a C57/BL6J background (stock no. 002561; The Jackson Laboratory) were housed in a temperature-controlled animal facility (21°C, 60% ± 5% humidity) on a 12-hour light/12-hour dark cycle and were allowed free access to autoclaved standard rodent chow (2018 Rodent Laboratory Chow; Harlan) and drinking water (tap water). Genotyping was performed according to the protocol provided by the supplier using tail genomic DNA. Only males were analyzed, as *jck* mice show sexual dimorphism in disease progression, with more aggressive disease in male mice. For the preparation of WT and *jck* MEFs, embryos of the corresponding genotype were identified at E13.5 using yolk sac genomic DNA, and MEFs were isolated as previously described (86).

### Generation of jck eIF2α^+/SA^ mice.

*eIF2α^+/SA^* female mice on a C57BL/6J background were crossed with *Nek8^jck/jck^* (*jck*) males to obtain *Nek8^+/jck^* *eIF2α^+/SA^* offspring. Male and female *Nek8^+/jck^* *eIF2α^+/SA^* offspring were then mated to obtain *jck* *eIF2α^+/SA^* male mice. The primers for the *eIF2α^SA^* mutation selection were as follows: forward: CACACACCCATTCCATGATAGTAAAAT and reverse: CAATGTTGTAGACCCTGACAA-TGAAGG, as previously reported (38).

### Antibodies.

The following antibodies were used for IHC and Western immunoblotting: ATF4 (catalog 10835-1-AP) and SKP2 (catalog 15010-1-AP) from Proteintech; CHOP (catalog 2895), p-YAP (catalog 13008, S127), YAP (catalog 14074), p27 (catalog 3686), MYC (catalog 9402), p-RPS6 (catalog 4858, S235/236), t-RPS6 (catalog 2217), p-mTORC1 (catalog 5536, S2448), t-mTORC1 (catalog 2983), p-ERK1/2 (catalog 9101, T202/Y204), t-eIF2α (catalog 2103), p-AKT (catalog 9271, S473), t-AKT (catalog 4691), EIF4FBP1 (catalog 2855, T37/46), K48-Ub (catalog 8081), p-PERK (catalog 3179, T980), and PERK (catalog 3192) from Cell Signaling Technology; CYR61 (catalog E-AB-14920), CTGF (E-AB-12339), and aquaporin 2 (E-AB-30540) from Elabscience Biotechnology; CDK1 (catalog SC-54) and t-ERK1/2 (catalog SC-94) from Santa Cruz Biotechnology; α-tubulin (catalog T5168) and K63-Ub (catalog 05-1308) from MilliporeSigma; PCNA (catalog MS-106-P1ABX and, p-LATS1/2 (catalog PA5-64591, S809/S872) from Thermo Fisher Scientific; Oct4 (catalog NB100-2379) from Novus Biologicals; Sox2 (catalog ab97959), vimentin (catalog ab92547), Ki-67 (clone SP6), p-eIF2α (catalog ab32157, S51), BIM (catalog ab32158), and TAZ (catalog ab224239) from Abcam; GRP78 (catalog A0241) from StressMarq Biosciences; and nestin 1 (monoclonal rat-401s) from DSHBU Iowa.

### Peptides and chemicals.

Forskolin (BioShop Canada) was used at a concentration of 10 μM. AVP was purchased from MilliporeSigma and used at a 10 nM concentration. SKP2 inhibitor (SKPin C1, S8652) was purchased from Selleckchem and was administered as a single intraperitoneal injection (20 mg/kg) to 1-month-old *jck* mice. Animals were euthanized 1 week later, and kidneys were harvested for analysis.

### Laser-scanning confocal fluorescence microscopy.

Paraffin tissue sections were deparaffinized, hydrated, and permeabilized using PBST (PBS containing 0.5% Triton X-100). Normal horse serum (Vector Laboratories) was used for blocking. Fluorescence labeling was done with anti–YAP Alexa Fluor 594 (Santa Cruz Biotechnology, sc-271134) and anti–Ub (linkage-specific K63) Alexa Fluor 488 (EPR8590-448; Abcam, ab192539) antibodies at a concentration of 1 μg/mL for both. Images were acquired using an LSM 800 Laser-Scanning Confocal Microscope (Carl Zeiss) under ×20 magnification. Pearson’s correlation coefficient was calculated with Volocity USA image analysis software (version 6.5.1).

### Treatment of jck mice with TUDCA or tolvaptan.

TUDCA and tolvaptan were purchased from Best of Chemicals Sciences (USA). To verify structure and purity, NMR and liquid chromatography–mass spectrometry (LC-MS) analysis was performed at the Drug Discovery Platform in the Research Institute of the McGill University Health Center (>98% pure). For chow preparation, tolvaptan was first suspended in 0.5% hydroxypropyl cellulose (0.5%), followed by sonication for 20 minutes to obtain an emulsified form because of its very low water solubility, and was then added (0.1% wt/wt) to grounded chow powder (Teklad, Envigo). Following homogeneous mixing, the chow was cut into appropriate-sized pieces and thoroughly dried before administration. TUDCA was dissolved in water, added to grounded chow powder (0.2% wt/wt), and processed similarly. Male *jck* mice, starting at 1 month of age, were fed either regular chow or tolvaptan- or TUDCA-containing chow for 4 consecutive days, followed by regular chow for the remaining 3 days of the week for 2 months. This treatment schedule avoided the attrition of animals observed when drug-containing chow was administered daily. At the end of 2 months (mice were 3 months old), ultrasonography was performed, and serum and kidneys were procured for analysis.

### Statistics.

All data are presented as the mean ± SEM. An unpaired Student’s *t* test was used for comparisons of 2 groups. One-way ANOVA with a Tukey-Kramer multiple-comparison test was used for comparisons of more than 2 groups. Statistical differences with a *P* value of less than 0.05 were considered significant.

### Study approval.

All animal procedures were reviewed and approved by the McGill University’s Institutional Animal Care Committee (protocols JGH-8121 to ACK and JGH-5754 for AEK). The mice were maintained in accordance with university guidelines for the care and use of laboratory animals.

## Author contributions

DKP, ACK, and MLL conceived the study. DKP, XB, YZ, and NAS conducted the experiments and acquired the data. DKP, ACK, and MLL analyzed the data. AEK contributed to work with eIF2αS52A mice and provided reagents and invaluable scientific input. DKP and ACK wrote the manuscript. All authors reviewed and approved the final version of the manuscript.

## Supplementary Material

Supplemental data

## Version 1 11/03/2022

In-Press Preview

## Version 2 12/15/2022

Electronic publication

## References

[1] Fry AM (2014). The primary cilium: guardian of organ development and homeostasis. Organogenesis.

[2] Praetorius HA, Spring KR (2003). Removal of the MDCK cell primary cilium abolishes flow sensing. J Membr Biol.

[3] Devlin LA, Sayer JA (2019). Renal ciliopathies. Curr Opin Genet Dev.

[4] Hughes J (1995). The polycystic kidney disease 1 (PKD1) gene encodes a novel protein with multiple cell recognition domains. Nat Genet.

[5] Mochizuki T (1996). PKD2, a gene for polycystic kidney disease that encodes an integral membrane protein. Science.

[6] Koulen P (2002). Polycystin-2 is an intracellular calcium release channel. Nat Cell Biol.

[7] Liang G (2008). Polycystin-2 down-regulates cell proliferation via promoting PERK-dependent phosphorylation of eIF2alpha. Hum Mol Genet.

[8] Pakos-Zebrucka K (2016). The integrated stress response. EMBO Rep.

[9] Costa-Mattioli M, Walter P (2020). The integrated stress response: from mechanism to disease. Science.

[10] Muller RU, Schermer B (2019). Hippo signaling-a central player in cystic kidney disease?. Pediatr Nephrol.

[11] Wong JS (2016). Hippo signaling in the kidney: the good and the bad. Am J Physiol Renal Physiol.

[12] Garcia-Garcia M (2022). Mechanical control of nuclear import by Importin-7 is regulated by its dominant cargo YAP. Nat Commun.

[13] Trudel M (1991). C-myc as an inducer of polycystic kidney disease in transgenic mice. Kidney Int.

[14] Cai J (2018). A RhoA-YAP-c-Myc signaling axis promotes the development of polycystic kidney disease. Genes Dev.

[15] Dwivedi N (2020). Epithelial vasopressin type-2 receptors regulate myofibroblasts by a YAP-CCN2-dependent mechanism in polycystic kidney disease. J Am Soc Nephrol.

[16] Happe H (2011). Altered Hippo signalling in polycystic kidney disease. J Pathol.

[17] Formica C (2020). Reducing YAP expression in Pkd1 mutant mice does not improve the cystic phenotype. J Cell Mol Med.

[18] Lemos FO, Ehrlich BE (2018). Polycystin and calcium signaling in cell death and survival. Cell Calcium.

[19] Ma S, Guan KL (2018). Polycystic kidney disease: a Hippo connection. Genes Dev.

[20] Kim H, Hwang YH (2016). Clinical trials and a view toward the future of ADPKD. Adv Exp Med Biol.

[21] Torres VE (2012). Tolvaptan in patients with autosomal dominant polycystic kidney disease. N Engl J Med.

[22] Torres VE (2017). Tolvaptan in later-stage autosomal dominant polycystic kidney disease. N Engl J Med.

[23] Liu S (2002). A defect in a novel Nek-family kinase causes cystic kidney disease in the mouse and in zebrafish. Development.

[24] Moniz L (2011). Nek family of kinases in cell cycle, checkpoint control and cancer. Cell Div.

[25] Sabbagh Y (2012). Repression of osteocyte Wnt/β-catenin signaling is an early event in the progression of renal osteodystrophy. J Bone Miner Res.

[26] Smith LA (2006). Development of polycystic kidney disease in juvenile cystic kidney mice: insights into pathogenesis, ciliary abnormalities, and common features with human disease. J Am Soc Nephrol.

[27] Frank V (2013). Mutations in NEK8 link multiple organ dysplasia with altered Hippo signalling and increased c-MYC expression. Hum Mol Genet.

[28] Devuyst O (1996). Expression of aquaporins-1 and -2 during nephrogenesis and in autosomal dominant polycystic kidney disease. Am J Physiol.

[29] Gabow PA (1989). The clinical utility of renal concentrating capacity in polycystic kidney disease. Kidney Int.

[30] Ewings KE (2007). Bim and the pro-survival Bcl-2 proteins: opposites attract, ERK repels. Cell Cycle.

[31] Woo D (1995). Apoptosis and loss of renal tissue in polycystic kidney diseases. N Engl J Med.

[32] Harding HP (1999). Protein translation and folding are coupled by an endoplasmic-reticulum-resident kinase. Nature.

[33] Casas C (2017). GRP78 at the centre of the stage in cancer and neuroprotection. Front Neurosci.

[34] Schroder M, Kaufman RJ (2005). ER stress and the unfolded protein response. Mutat Res.

[35] Yamaguchi H, Wang HG (2004). CHOP is involved in endoplasmic reticulum stress-induced apoptosis by enhancing DR5 expression in human carcinoma cells. J Biol Chem.

[36] Wu H (2015). Integration of Hippo signalling and the unfolded protein response to restrain liver overgrowth and tumorigenesis. Nat Commun.

[37] Grampa V (2016). Novel NEK8 mutations cause severe syndromic renal cystic dysplasia through YAP dysregulation. PLoS Genet.

[38] Scheuner D (2001). Translational control is required for the unfolded protein response and in vivo glucose homeostasis. Mol Cell.

[39] Dalgaard OZ (1957). Bilateral polycystic disease of the kidneys; a follow-up of two hundred and eighty-four patients and their families. Acta Med Scand Suppl.

[40] Torres VE (2006). Water for ADPKD? Probably, yes. J Am Soc Nephrol.

[41] Koromilas AE (2019). M(en)TORship lessons on life and death by the integrated stress response. Biochim Biophys Acta Gen Subj.

[42] Park Y (2017). mTORC1 balances cellular amino acid supply with demand for protein synthesis through post-transcriptional control of ATF4. Cell Rep.

[43] Chen YJ (2010). Differential regulation of CHOP translation by phosphorylated eIF4E under stress conditions. Nucleic Acids Res.

[44] Rajesh K (2016). The eIF2α serine 51 phosphorylation-ATF4 arm promotes HIPPO signaling and cell death under oxidative stress. Oncotarget.

[45] Yao F (2018). SKP2- and OTUD1-regulated non-proteolytic ubiquitination of YAP promotes YAP nuclear localization and activity. Nat Commun.

[46] Pickart CM, Fushman D (2004). Polyubiquitin chains: polymeric protein signals. Curr Opin Chem Biol.

[47] Yang WH (2021). The Hippo pathway effector YAP promotes ferroptosis via the E3 ligase SKP2. Mol Cancer Res.

[48] Abukhdeir AM, Park BH (2008). P21 and p27: roles in carcinogenesis and drug resistance. Expert Rev Mol Med.

[49] Dupont S (2011). Role of YAP/TAZ in mechanotransduction. Nature.

[50] Praetorius HA, Spring KR (2001). Bending the MDCK cell primary cilium increases intracellular calcium. J Membr Biol.

[51] Hirose K (1999). Spatiotemporal dynamics of inositol 1,4,5-trisphosphate that underlies complex Ca2+ mobilization patterns. Science.

[52] Koslowski S (2020). An overview of in vivo and in vitro models for autosomal dominant polycystic kidney disease: a journey from 3D-cysts to mini-pigs. Int J Mol Sci.

[53] Valentich JD (1979). Hemicyst formation stimulated by cyclic AMP in dog kidney cell line MDCK. J Cell Physiol.

[54] Mangoo-Karim R (1989). Renal epithelial cyst formation and enlargement in vitro: dependence on cAMP. Proc Natl Acad Sci U S A.

[55] Liu-Chittenden Y (2012). Genetic and pharmacological disruption of the TEAD-YAP complex suppresses the oncogenic activity of YAP. Genes Dev.

[56] Engin F (2013). Restoration of the unfolded protein response in pancreatic β cells protects mice against type 1 diabetes. Sci Transl Med.

[57] Kusaczuk M (2019). Tauroursodeoxycholate-bile acid with chaperoning activity: molecular and cellular effects and therapeutic perspectives. Cells.

[58] Wang X (2008). Vasopressin directly regulates cyst growth in polycystic kidney disease. J Am Soc Nephrol.

[59] Hopp K (2015). Tolvaptan plus pasireotide shows enhanced efficacy in a PKD1 model. J Am Soc Nephrol.

[60] Martinez-Maldonado M (1972). Adult polycystic kidney disease: studies of the defect in urine concentration. Kidney Int.

[61] Lin HK (2009). Phosphorylation-dependent regulation of cytosolic localization and oncogenic function of Skp2 by Akt/PKB. Nat Cell Biol.

[62] Wu L (2012). Specific small molecule inhibitors of Skp2-mediated p27 degradation. Chem Biol.

[63] Zhao H (2019). SKP2 targeted inhibition suppresses human uveal melanoma progression by blocking ubiquitylation of p27. Onco Targets Ther.

[64] Happe H, Peters DJ (2014). Translational research in ADPKD: lessons from animal models. Nat Rev Nephrol.

[65] Piontek K (2007). A critical developmental switch defines the kinetics of kidney cyst formation after loss of Pkd1. Nat Med.

[66] Nishimura Y (2019). Primary cilia as signaling hubs in health and disease. Adv Sci (Weinh).

[67] Zhou X (2021). PKD2 deficiency suppresses amino acid biosynthesis in ADPKD by impairing the PERK-TBL2-eIF2ɑ-ATF4 pathway. Biochem Biophys Res Commun.

[68] Winyard P, Jenkins D (2011). Putative roles of cilia in polycystic kidney disease. Biochim Biophys Acta.

[69] Formica C, Peters DJM (2020). Molecular pathways involved in injury-repair and ADPKD progression. Cell Signal.

[70] Dibble CC, Cantley LC (2015). Regulation of mTORC1 by PI3K signaling. Trends Cell Biol.

[71] Torrence ME (2021). The mTORC1-mediated activation of ATF4 promotes protein and glutathione synthesis downstream of growth signals. Elife.

[72] Carrano AC (1999). SKP2 is required for ubiquitin-mediated degradation of the CDK inhibitor p27. Nat Cell Biol.

[73] Asmamaw MD (2020). Skp2 in the ubiquitin-proteasome system: a comprehensive review. Med Res Rev.

[74] Cai Z (2020). The Skp2 pathway: a critical target for cancer therapy. Semin Cancer Biol.

[75] Chan CH (2012). The Skp2-SCF E3 ligase regulates Akt ubiquitination, glycolysis, herceptin sensitivity, and tumorigenesis. Cell.

[76] Geng Q (2017). Phosphorylation by mTORC1 stablizes Skp2 and regulates its oncogenic function in gastric cancer. Mol Cancer.

[77] Jang W (2017). Mechanical cue-induced YAP instructs Skp2-dependent cell cycle exit and oncogenic signaling. EMBO J.

[78] Seeger-Nukpezah T (2015). The hallmarks of cancer: relevance to the pathogenesis of polycystic kidney disease. Nat Rev Nephrol.

[79] Karami S (2016). Risk of renal cell carcinoma among kidney transplant recipients in the United States. Am J Transplant.

[80] Grantham JJ (1990). Polycystic kidney disease: neoplasia in disguise. Am J Kidney Dis.

[81] Woo YM (2016). Validation of effective therapeutic targets for ADPKD using animal models. Adv Exp Med Biol.

[82] Serra AL (2010). Sirolimus and kidney growth in autosomal dominant polycystic kidney disease. N Engl J Med.

[83] Walz G (2010). Everolimus in patients with autosomal dominant polycystic kidney disease. N Engl J Med.

[84] Vang S (2014). The unexpected uses of urso- and tauroursodeoxycholic acid in the treatment of non-liver diseases. Glob Adv Health Med.

[85] Natoli TA (2010). Inhibition of glucosylceramide accumulation results in effective blockade of polycystic kidney disease in mouse models. Nat Med.

[86] Durkin ME (2013). Isolation of mouse embryo fibroblasts. Bio Protoc.

