# Supplementary material for: SCF-SKP2 E3 ubiquitin ligase links mTORC1/ER stress/ISR with YAP activation in murine renal cystogenesis
Source: J Clin Invest. 2022 Dec 15;132(24):e153943. doi: 10.1172/JCI153943 (PMC9754004; doi:10.1172/JCI153943)
Supplement: Supplemental data [file jci-132-153943-s047.pdf]

SCF-SKP2 E3 ubiquitin ligase links mTORC1-ER stress-ISR with YAP activation in murine renal cystogenesis

Dibyendu K. Panda[1,2], Xiuying Bai[1], Yan Zhang[2], Nicholas A. Stylianesis[3], Antonis E. Koromilas[4,5], Mark L. Lipman[2], Andrew C. Karaplis[1]

[1] Division of Endocrinology and Metabolism, Department of Medicine, and Lady Davis Institute for Medical Research, Jewish General Hospital, McGill University, Montréal, Québec, Canada

[2] Division of Nephrology, Department of Medicine, and Lady Davis Institute for Medical Research, Jewish General Hospital, McGill University, Montréal, Québec, Canada

[3] Department of Chemical Engineering, McGill University, Montréal, Québec, Canada

[4] Gerald Bronfman Department of Oncology, Faculty of Medicine, McGill University, Montréal, Québec, Canada

[5] Lady Davis Institute for Medical Research, Jewish General Hospital, Montréal, Québec, Canada

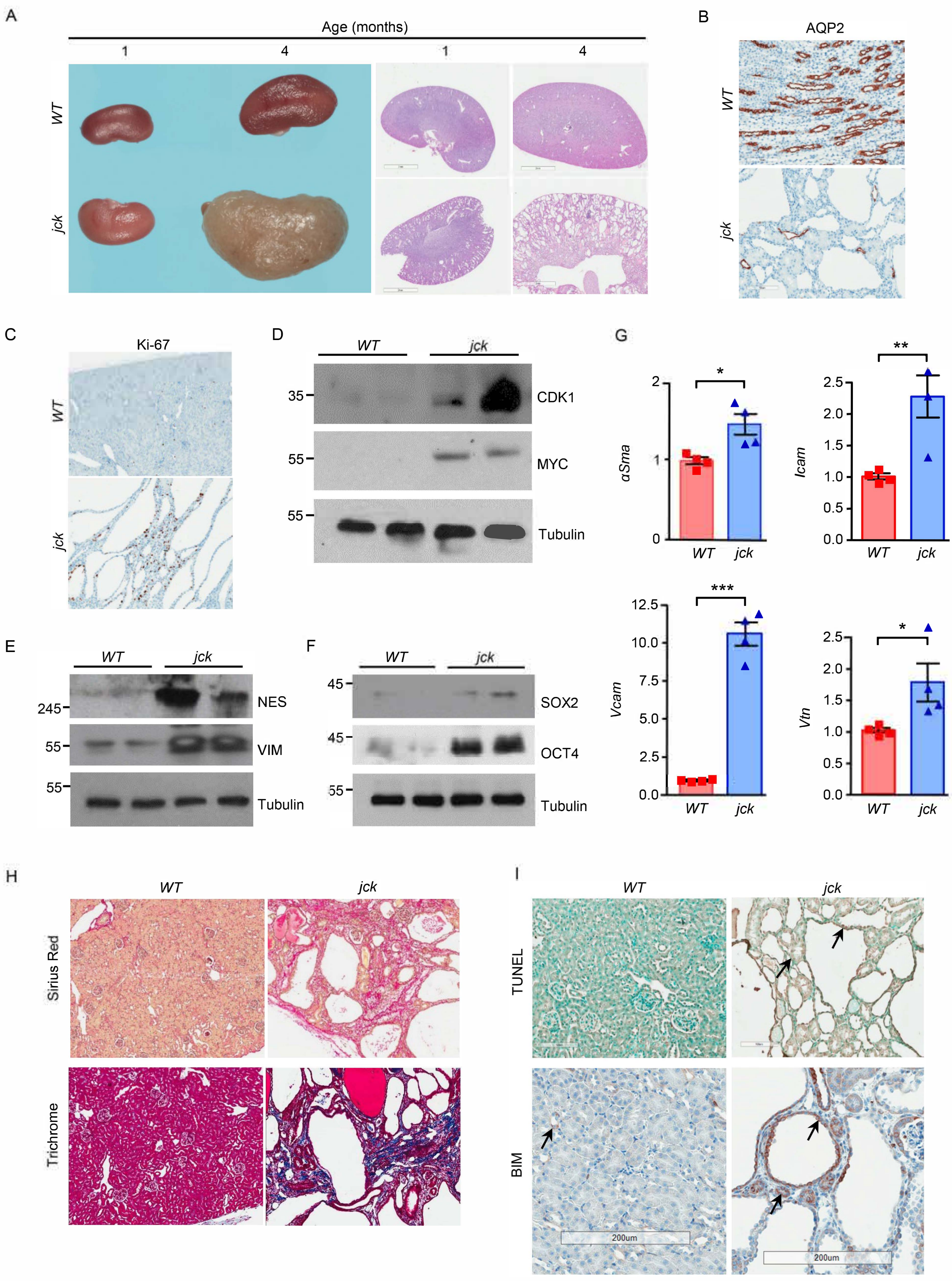

Supplemental Figure 1

Supplemental figures

Supplemental Figure 1. The *jck* PKD phenotype. (**A**) Representative gross and H&E-stained histologic images of kidneys from *jck* and *WT* mice at 1 and 4 months of age. Scale bars: 2 mm. At 4 months of age, (**B**) Aquaporin 2 (AQP2), and (**C**) Ki67 expression by immunohistochemistry. Scale bars: 100 μm. (**D**) CDK1 and MYC expression by Western blotting. Expression of molecular markers of (**E**) renal interstitial damage, [NES (Nestin), VIM (Vimentin)], and (**F**) cellular dedifferentiation (SOX2, OCT4), by western immunoblotting, and (**G**) epithelial-mesenchymal transition and tissue inflammation [α*Sma, Vcam, Icam,* and *Vtn* (*Vitronectin*)] by quantitative real-time PCR, relative to *β-Actin* (*WT*=4, *jck*=4). (**H**) Collagen deposition (Sirius Red), and tissue fibrosis (Trichrome), as determined by histochemistry. (**I**) Apoptosis of renal tubular epithelial cells (arrows) by TUNEL and BIM immunostaining. Data represent mean ± SEM. Differences between samples were analyzed for statistical significance by Student's unpaired *t*-test; *$P<0.05$, **$P<0.01$, ***$P<0.001$. Data are representative of at least two independent experiments.

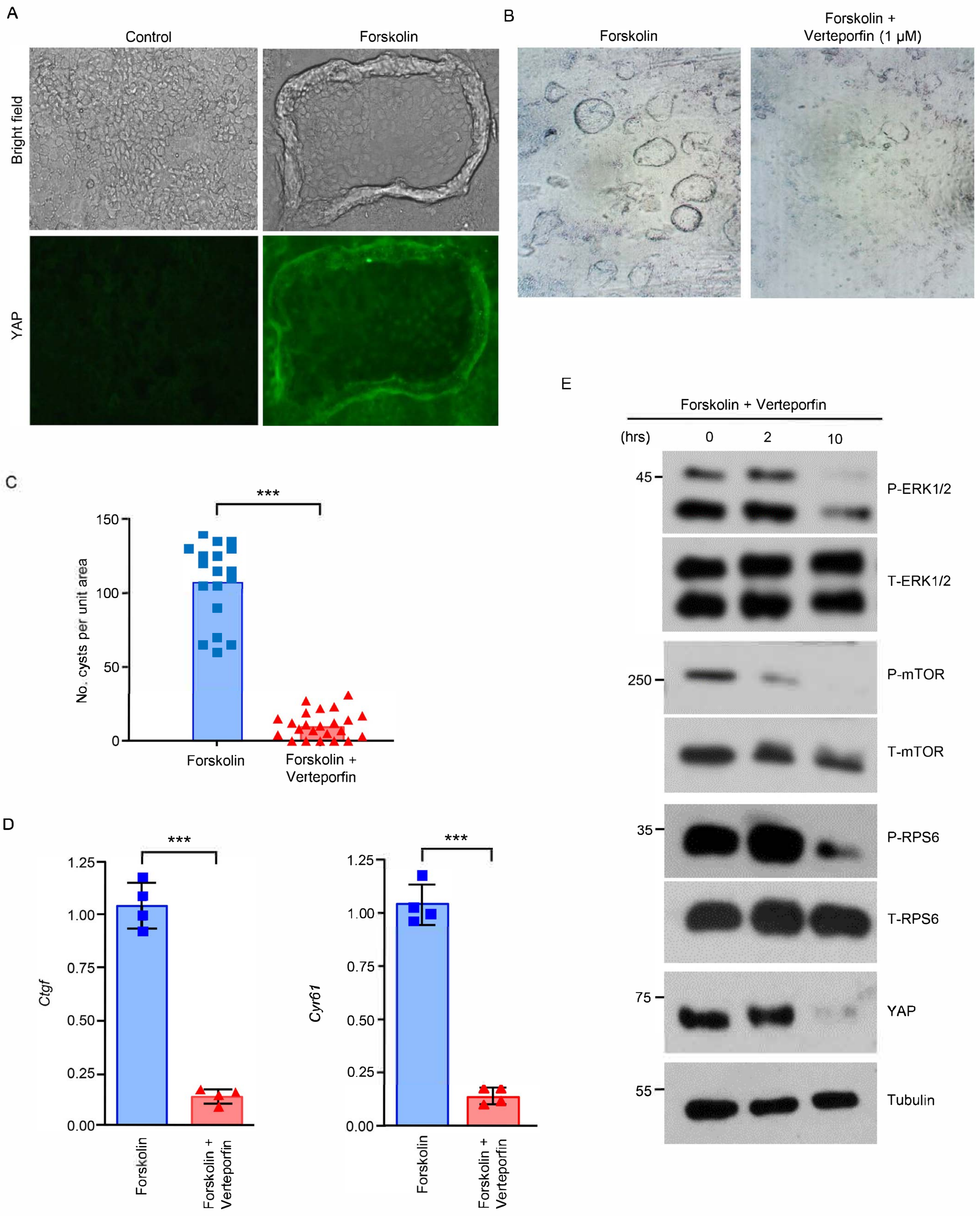

Supplemental Figure 2

Supplemental Figure 2. YAP promotes cystogenesis in vitro. (**A**) Bright-field and YAP immunofluorescence of cultured MDCKII cells in the absence (left panels) and presence (right panels) of forskolin (10 μM). (**B**) Cyst-like development by forskolin-treated MDCKII cells in the absence and presence of the YAP-TEAD inhibitor, verteporfin (1 μM). (**C**) Number of cysts per unit area counted (n=18 for forskolin group; n=23 for verteporfin group). Data are representative of two independent experiments. (**D**) YAP/TEAD target gene expression (*Ctgf* and *Cyr61*) following forskolin treatment of MDCKII cells in the absence and presence of verteporfin. (**E**) Addition of verteporfin to forskolin-treated MDCKII cells (4 hrs pretreatment) decreased levels of P-ERK1/2, P-mTOR, P-RPS6, and YAP while levels of T-ERK1/2, T-mTOR, and T-RPS6 were unchanged. Data represent mean ± SEM. Differences between samples were analyzed for statistical significance by Student's unpaired *t*-test; ***$P<0.001$.

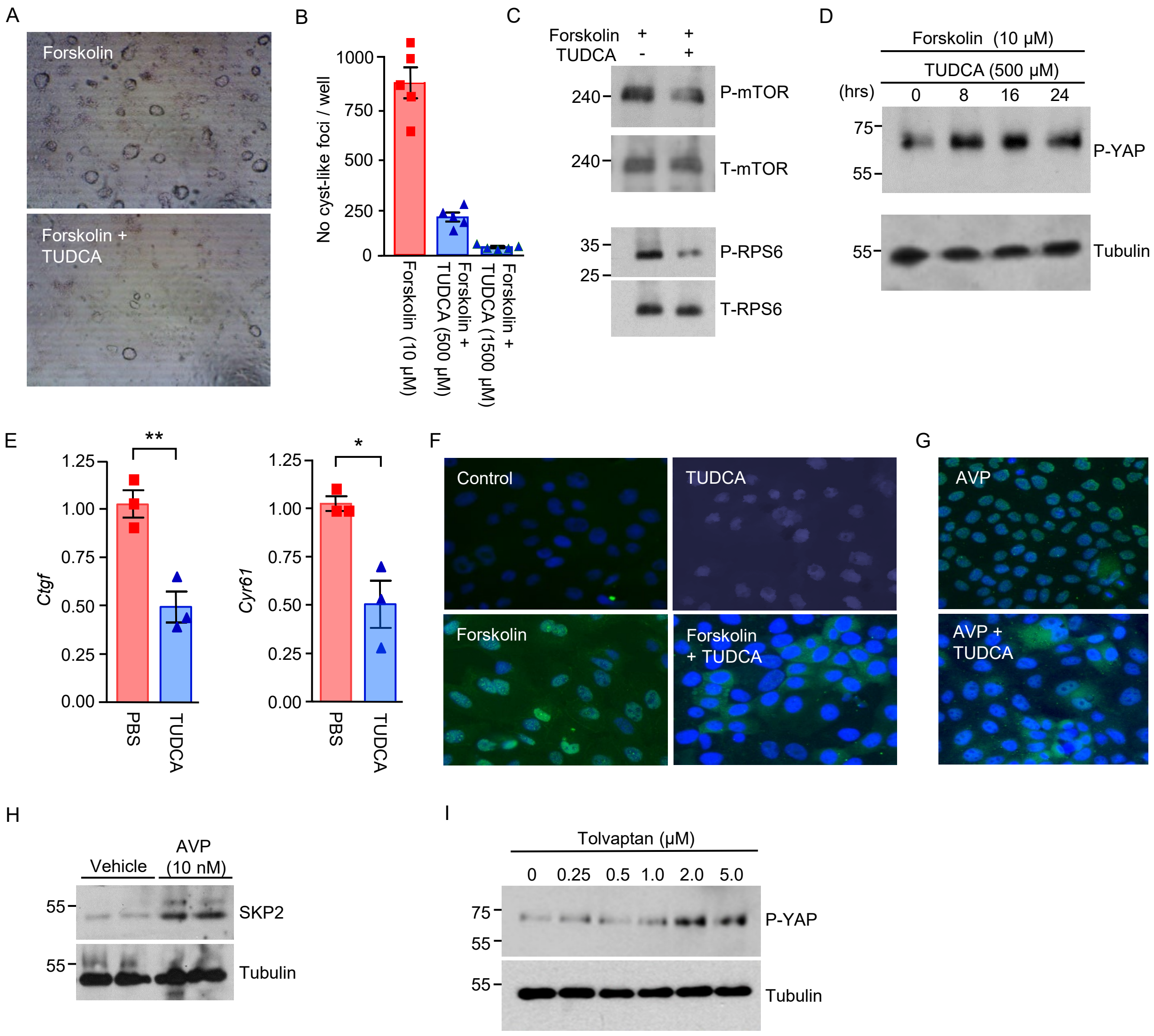

Supplemental Figure 3

Supplemental Figure 3. Relief of ER stress impairs YAP activity in vitro. (**A**) Cyst-like development by forskolin-treated MDCKII cells in the absence and presence of TUDCA (500 μM). (**B**) TUDCA prevents cyst-like formation in vitro in a concentration-dependent fashion. (**C**) TUDCA treatment decreased levels of P-mTOR and P-RPS6 in response to forskolin. (**D**) TUDCA treatment increased levels of P-YAP. (**E**) Quantitative real-time PCR analysis showing *Ctgf* and *Cyr61* expression in MDCKII cells following TUDCA treatment, using *β-Actin* as internal control. (**F**) YAP fluorescence microscopy (green immunofluorescence) in MDCKII cells localizing it to the nucleus when cells were treated with forskolin (10 μM) for 18 hours. The addition of TUDCA (500 μM) shifted the localization of endogenous YAP immunofluorescence to the cytoplasm. DAPI blue-fluorescent DNA stain was used as a nuclear counterstain. (**G**) YAP immunofluorescence in MDCKII cells localizing to the nucleus when cells were treated with AVP (10 nM) for 18 hours. The addition of TUDCA (500 μM) shifted YAP immunofluorescence to the cytoplasm. DAPI blue-fluorescent DNA stain was used as a nuclear counterstain. (H) AVP treatment (10 nM) of MDCKII cells increased SKP2 expression. (**I**) Increasing concentrations of tolvaptan raised levels of P-YAP in MDCKII cells. Data represent mean ± SEM. Differences between groups were analyzed for statistical significance by Student's unpaired *t*-test; *$P<0.05$, **$P<0.01$. Data are representative of two independent experiments.

Supplemental methods

Kidney ultrasonography. Mice were anesthetized with isoflurane, the fur was removed with depilatory cream, and ultrasound Eco gel 100 was applied. The VEVO3100 Ultra High-Frequency ultrasound system was used to obtain 2D images of the kidneys in B-mode and acquired data were used to reconstruct 3D images of the organ. Kidney volume was determined from the reconstructed 3D images. For cyst analysis, uniformity was maintained between the samples by choosing the 2D image for each kidney sample where the renal artery can be distinctly identified. The diameter of individual cysts was determined, the area was calculated, and the statistical significance was determined. Data were analyzed using the VEVO3100 software.

Urine collection/analysis. Urine was collected from individual animals from each treatment group one week before euthanasia. Mice were placed individually in metabolic cages with only access to water for 4 hours. Urine volumes were recorded and stored at -80°C freezer before osmolarity measurements. Osmolality was measured using Advanced Instruments Model 2020 Micro Osmometer. Calibration was done with solutions of 2000 mOsm/kg to 50 mOsm/kg, and a reference solution of 290 mOsm/kg.

Gene expression. RNA was isolated from the tissue samples using TRIZOL reagent (Invitrogen Inc). cDNA was synthesized using TranScript one-in-all kit (TransGen Biotech, Inc), and gene expression was measured by quantitative real-time PCR using a LightCycler FastStartDNA Master SYBR Green 1 kit (Roche). Relative gene expression was normalized against *β-Actin* expression. The set of primers used to analyze gene expression are outlined below:

Vcam1-F TCTCTCAGGAAATGCCACCC	*Vcam1*-R CACAGCCAATAGCAGCACAC;
Icam1-F AACTTTTCAGCTCCGGTCCTG	*Icam1*-R TCAGTGTGAATTGGACCTGCG;
αSma-F ACTGGGACGACATGGAAAAG	*αSma*-R CATCTCCAGAGTCCAGCACA;
Vtn-F GCCGGGAAAGGGTCTACTT	*Vtn*-R TGCTGCTGAAATTCGTACTCC;
Ctgf-F GCTGACCTGGAGGAACATTAA	*Ctgf*-R TGACAGGCTTGGCGATTTTAG;
Cyr61-F CCTTCTCCACTTGACCAGAC	*Cyr61*-R ATATTCACAGGGTCTGCCTTCT;
β-Actin-F AAGGCCAACCGTGAAAAGAT	*β-Actin*-R GTGGTACGACCAGAGGCATAC;
Myc-F GCGACTCTGAAGAAGAGCAAG	*Myc*-R GCCTCGGGATGGAGATGAG ;
Nppb-F TCCTAGCCAGTCTCCAGAGCAA	*Nppb*-R GGTCCTTCAAGAGCTGTCTCTG;
Ankrd1-F GCTTAGAAGGACACTTGGCGATC	*Ankrd1*-R GACATCTGCGTTTCCTCCACGA;
Taz1-F CCTTATCACCGTCTCCAACCAC	*Taz1*-R CCTTGGTGAAGCAGATGTCTGC

In vitro cyst-like formation. MDCKII cells (generously provided by Dr. S. Lemay, McGill University) were cultured in a mixture of DMEM and Ham's F12 (1:1) nutrient medium containing 10% FBS, 100 U/ml of penicillin, and 100 µg/ml of streptomycin. In vitro 2D cyst-like formation was performed by plating the cells in the presence of 10 µM forskolin. The culture medium was replenished every 3 days for 14 days. Images were taken using light microscopy. To study the effect of verteporfin (1 µM), TUDCA (500 µM), and AVP (10 nM) the culture medium was supplemented with each of these compounds at the indicated concentrations, in addition to forskolin. For the verteporfin experiments, cells were protected from light at all times.

Chloral hydrate treatment - MDCKII cells were grown in 6 well tissue culture dishes in DMEM/F12 medium containing 10% FCS. After 24 hours, cells were starved in DMEM/F12 medium containing 1% FCS for 24 hours and then treated with vehicle (ethanol) or with varying concentrations of chloral hydrate (0.5, 1, 2, and 4 mM) for 18 hours. Cells were then lysed in RIPA buffer containing protease inhibitor (cOmplete protease inhibitor cocktail, Millipore-Sigma) and 10 µg of total protein was separated in 10% polyacrylamide gel, and transferred to nitrocellulose membrane. Levels of protein expression was analysed by western blotting and the intensity of the bands was measured by ImageJ software.

Parallel-plate flow chamber (PPFC) setup - MDCKII cells were subjected to a constant shear stress of 3 dyn/cm^2. This was achieved by setting up a flow system in which media supplied by an in-circuit reservoir was transported by a peristaltic pump into a parallel-plate flow chamber containing cells. A Cole Parmer flow dampener (Masterflex – Item # UZ-07596-20) was also introduced serially into the system so that more than 90% of the energy associated with the pulsatile flow was lost to the volume within the dampener. Algebraic analysis showed that to achieve the desired 3 dyn/cm^2, the flow rate required was 0.22 mL/s and this translated to 16 RPMs for the peristaltic pump.

Yap immunofluorescence - MDCKII cells or *WT* and *jck* MEFs were grown in 4-well chamber slides. After 24 hours, MDCKII cells and *WT* MEFs were treated either with vehicle or with 4 mM of chloral hydrate for 18 hours. All three cell types were then fixed in PLP solution containing 4% paraformaldehyde and L-Lysine. Immunofluorescence was performed using a rabbit anti-YAP antibody (Cell Signaling

14074) as primary and Alexa Fluor 488 (FITC-labeled) goat anti-rabbit IgG (Molecular Probe, Invitrogen) as secondary antibody, while DAPI (Invitrogen) fluorescent staining was used to delineate the nuclei. Cellular distribution of YAP (green staining) was compared with the blue staining of DAPI and images were captured using a Leica DFC350FX microscope.

Immunoprecipitation. Cell lysates was prepared from kidney samples by dissolving in NP-40 lysis buffer (50 mM Tris pH 7.4, 250 mM NaCl, 5 mM EDTA, 1% NP-40 and 0.02% sodium azide), protease inhibitor cocktail (Roche) supplemented with 0.2 mM PMSF, 1 mM beta-glycerophosphate, 1 mM sodium vanadate, and 10 nM MG132. 100 µg of total cell lysate were mixed with primary antibody (YAP 8J19 DSHB), at appropriate dilution, and incubated at 4°C for 4 hours using a rotator. 50 µl of protein A agarose (50% slurry) was added and incubation continued at 4˚C overnight. Immunoprecipitated complexes were collected by centrifugation at 3000xg for 5 min at 4°C and the supernatant was discarded. A washing buffer (same as the lysis buffer) was used to wash the immunoprecipitate thrice which was subsequently dissolved in the loading buffer for SDS-PAGE analysis.

Histology and immunohistochemistry. Kidney samples were procured, fixed in PLP fixative (2% paraformaldehyde containing 0.075 M lysine and 0.01 M sodium periodate solution) for 24h at 5°C, and embedded in paraffin. Tissue samples were cut at 4-µm thickness, placed on SuperFrost/Plus slides (Fisher), and dried overnight at 37°C, before processing. Immunohistochemistry was performed at the Segal Cancer Centre Research Pathology Facility (Jewish General Hospital, Montreal). Sections were deparaffinized, conditioned, and loaded onto the Discovery XT

Autostainer (Ventana Medical System) for antigen retrieval. All solutions used for automated immunohistochemistry were from the Ventana Medical System.

Immunofluorescence. Immunofluorescence was performed at the Segal Cancer Centre Research Pathology Facility (Jewish General Hospital, Montreal). Tissue samples were cut at 4-μm thickness, placed on SuperFrost/Plus slides (Leica), and dried overnight at 37°C, before staining. Primary antibodies for YAP and K63-Ub were applied to the tissue for 30 min. Fully automated 5-plex fluorescent immunohistochemistry with tyramide signal amplification and same species antibodies was performed as described (96). A negative control was performed by the omission of the primary antibodies. Slides were removed from the autostainer, washed, and counterstained with DAPI for five minutes, then mounted with a mounting medium (Immu-Mount, Thermo-Scientific). Sections were imaged with the appropriate fluorescent filter sets using Nikon Upright motorized microscope.

Western blot analysis. 10-15 μg total protein was loaded in each slot, separated by electrophoresis, proteins transferred to a nitrocellulose membrane, and immunoblotting was performed. The intensity of the bands was measured using ImageJ software.

Sirius red and trichrome staining. Sections were stained with picro-sirius red solution for 1 hr followed by brief exposure to acidified water. After washing, sections were counterstained with hematoxylin. Trichrome staining was performed by immersing the sections in Biebrich scarlet-acid fuchsin solution for 10-15 minutes, differentiated in phosphomolybdic-phosphotungstic acid solution for 10-15 minutes. Sections were transferred to aniline blue solution and finally differentiated in acetic acid solution

briefly. After washing in water, sections were dehydrated, cleared in xylene, and mounted.

Apoptosis assay. In situ labeling kit (Novus Biologicals) was used for the detection of apoptosis. Paraffin sections of kidney samples were deparaffinized, hydrated, and Br-dU-based end-labeling of DNA in the apoptotic cells was performed following the supplier's instructions.
